# Exploring the mechanism of Qinlian Yuyang Decoction in the treatment of ulcerative colitis based on multi-omics technology

**DOI:** 10.1186/s13020-026-01380-6

**Published:** 2026-03-20

**Authors:** Ya-ting Cao, Xin Huang, Cheng-li Yu, Jing Wang, Xue Han, Chen-wen Wang, Zi-chen Luo, Wei-chen Xu, Jin-jun Shan, Yong-ming Li, Kang Ding, Ye Zhang, Ai-ling Yin

**Affiliations:** 1 Medical Experimental Centre, Central Laboratory, Nanjing Hospital of Chinese Medicine Affiliated to Nanjing University of Chinese Medicine, Nanjing, 210022 Jiangsu China; 2 Department of Biobank, Nanjing Hospital of Chinese Medicine Affiliated to Nanjing University of Chinese Medicine, Nanjing, 210022 Jiangsu China; 3 Jiangsu Key Laboratory for Functional Substances of Chinese Medicine, School of Pharmacy, Nanjing University of Chinese Medicine, Nanjing, 210023 Jiangsu China; 4 State Key Laboratory of Oral Drug Delivery Systems of Chinese Materia Medica, Affiliated Hospital of Integrated Traditional Chinese and Western Medicine, Nanjing University of Chinese Medicine, Nanjing, 210028 Jiangsu China; 5 State Key Laboratory of Natural Medicines, School of Traditional Chinese Pharmacy, China Pharmaceutical University, Nanjing, 211198 Jiangsu China; 6 Institute of Pediatrics, Jiangsu Key Laboratory of Pediatric Respiratory Disease, Medical Metabolomics Center, Nanjing University of Chinese Medicine, Nanjing, 210023 Jiangsu China; 7 School of Medicine and Holistic Integrative Medicine, Nanjing University of Chinese Medicine, Nanjing, 210023 Jiangsu China; 8 National Center of Colorectal Surgery, Nanjing Hospital of Chinese Medicine Affiliated to Nanjing University of Chinese Medicine, Nanjing, 210001 Jiangsu China

**Keywords:** Qinlian Yuyang Decotion, Ulcerative colitis, Gut microbiota, *Dorea longicatena*, Tyrosine

## Abstract

**Background:**

The global incidence of ulcerative colitis (UC) is increasing, yet effective clinical treatment options remain limited. Qinlian Yuyang Decoction (QYD), a modified formulation based on Gegen Qinlian Decoction (GQD), has been optimized to better alleviate the pathological characteristics of UC. Although QYD is considered potentially more suitable for UC treatment, its potential mechanism of action is still unclear.

**Purpose:**

To explore the mechanism of QYD in the treatment of UC based on multi-omics.

**Method:**

The therapeutic effects of QYD for UC were evaluated using the dextran sulfate sodium (DSS)-induced UC mice model. Then, 16S rRNA sequencing and untargeted metabolomics were employed to identify key microbiota and metabolites regulating the intestinal microenvironment. Furthermore, proteomic analysis was carried out to elucidate the underlying mechanisms mediated by the key metabolites.

**Results:**

QYD alleviated physiochemical indices in UC mice, including weight loss, diarrhea, and bloody stools, resulting in lower Disease Activity Index (DAI) scores. It also suppressed inflammatory and intestinal barrier disruption in colonic tissues. Integrated metabolomic and 16S rRNA analyses demonstrated that the therapeutic effects of QYD on UC are mediated through enrichment of gut commensal *D. longicatena* and increasing tyrosine levels. Oral treatment of live *D. longicatena* considerably ameliorates colitis symptoms in UC mice. Tyrosine has similar protective effects against UC. Our investigation using quantitative proteomics to elucidate the mechanism of tyrosine in UC revealed that tyrosine suppresses inflammatory signaling pathways, modulates immune responses, and inhibits vascular endothelial cell migration and proliferation in the UC model—effects consistent with those of QYD. In summary, our findings indicate that QYD alleviates UC by elevating gut levels of the commensal bacterium *D. longicatena* and tyrosine, thereby suppressing inflammatory and immune responses while protecting the intestinal barrier. These results identify novel therapeutic targets and provide fresh perspectives for UC treatment.

**Conclusion:**

These findings suggest that *Dorea longicatena* and tyrosine play indispensable roles in the therapeutic mechanism of QYD against UC.

**Graphical Abstract:**

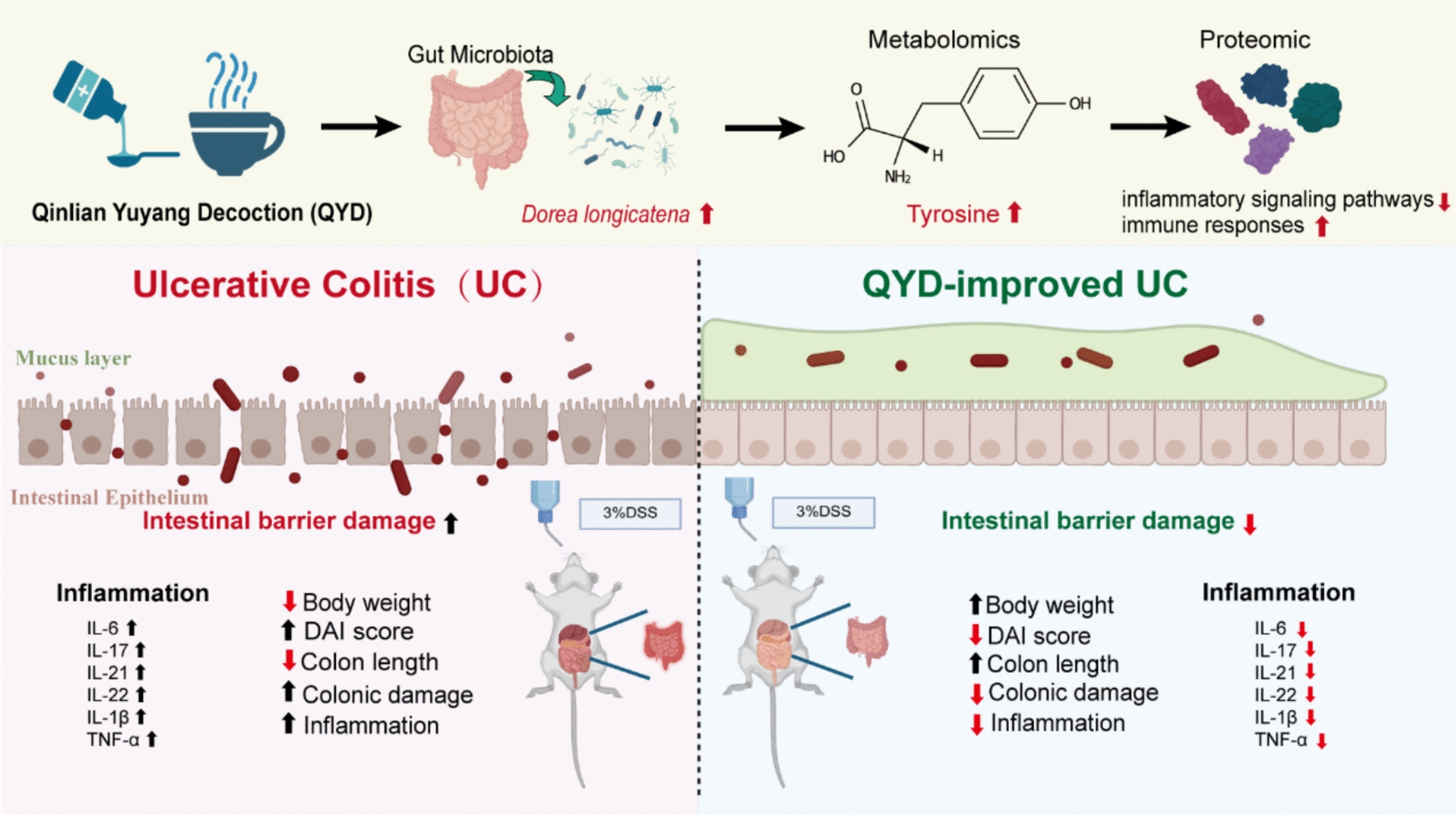

**Supplementary Information:**

The online version contains supplementary material available at 10.1186/s13020-026-01380-6.

## Introduction

UC is a chronic inflammatory bowel disease characterized by nonspecific inflammation of the colonic and rectal mucosa. The primary clinical manifestations include abdominal pain, and mucopurulent bloody stools. Secondary symptoms such as abdominal distension, anorexia, nausea, and vomiting are also common [1]. Recent epidemiological data indicate a marked increase in UC incidence worldwide. As the disease primarily affects young and middle-aged adults, its growing prevalence poses a substantial socioeconomic burden [2]. Although the precise etiology of UC remains unclear, it is generally attributed to a complex interplay among genetic susceptibility, environmental factors, immune dysregulation, and gut microbiota imbalance. The disease usually follows a relapsing–remitting course, imposing considerable physical and psychological strain on affected individuals. Current management primarily relies on pharmacotherapy with 5-aminosalicylic acid (5-ASA) derivatives, corticosteroids, immunosuppressants, and biologic agents. However, these treatments are frequently associated with significant adverse effects, underscoring the urgent need for safer and more effective therapeutic strategies [3].

The gut microbiota dysbiosis is one of an important pathogenetic mechanisms of UC [4, 5]. The gut microbiota contributes to UC pathogenesis through dynamic alterations in its metabolic profiles, whereas microbial dysbiosis may also serve as a potential biomarker of the disease. A decline in microbial diversity reduces species richness and undermines the stability of the intestinal ecosystem. Consequently, targeted modulation of the gut microbiota has emerged as a promising therapeutic strategy, with the potential to overcome the limitations of current treatments [6, 7].

Traditional Chinese medicine (TCM) provides novel therapeutic strategies for UC based on its unique theoretical framework [8], such as GQD, Baituoweng Decoction, Huanglian Jiedu Decoction, Shenling Baizhu Powder, and Zuojin Pills. GQD, a classical formulated by Zhang Zhongjing, consists of *Pueraria lobata, Scutellaria baicalensis, Coptis chinensis, and Glycyrrhiza uralensis*. It is primarily indicated for damp-heat diarrhea, and accumulating evidence suggests that GQD ameliorates UC by reshaping the gut microbial community [9]. QYD, a derivative of GQD developed through clinical experience and grounded in TCM theory, consists of *Pueraria lobata*, *Scutellaria baicalensis*, *Coptis chinensis*, *Glycyrrhiza uralensis*, *Paeonia lactiflora*, *Angelica sinensis*, *Sanguisorba officinalis*, *Portulaca oleracea*, *Aucklandia lappa*. It is customized to the specific clinical manifestations of UC, providing a more targeted therapeutic option. However, its precise mechanisms of action require further elucidation. Therefore, this study investigates the mechanism of QYD in the treatment of UC by through multi-omics analysis.

## Materials and methods

### Preparation of QYD

QYD herbal slices were decocted in distilled water at a 1:10 (w/v) ratio for 1 h. This extraction procedure was performed twice, and the resulting decoctions were combined. The pooled extract was filtered through several layers of gauze to remove residues and subsequently centrifuged. The supernatant was collected, freeze-dried to obtain a powder, and stored at -20 °C. For oral administration to mice, the lyophilized powder was reconstituted in 0.5% (w/v) sodium carboxymethylcellulose (CMC-Na) solution.

### Animal study

All animal procedures were approved by the Animal Ethics Committee of Nanjing University of Chinese Medicine (Approval No: AEWC-20250314-507) and were conducted in accordance with institutional guidelines. Healthy female C57BL/6 mice (6 weeks old, 18–20 g) were obtained from GemPharmatech Co., Ltd. (License No: SCXK(Su)2023-0009) and maintained under SPF conditions (21 ± 2 °C, 55–65% humidity, 12 h light/dark cycle) with ad libitum access to sterilized chow and water. After a one-week acclimation period, mice were randomly assigned to experimental groups.

For the QYD pharmacodynamic study, mice were divided into five groups (n = 6): Control, DSS, Low-dose QYD, High-dose QYD, and 5-ASA (positive control) [10]. Acute colitis was induced in all but the Control group by administering 3% DSS in drinking water for 7 days; the Control group received normal water [11]. All mice in the experimental groups were provided ad libitum access to the same irradiated sterilized feed, with a daily ration of 100 g per cage. The feed (designated XTI01CR-010 for laboratory mouse growth and reproduction) was supplied by Jiangsu Xietong Pharmaceutical Bio-Engineering Co.,Ltd. It contained 24.0% crude protein, with a phenylalanine + tyrosine content of 16.7 g/kg. This standardized diet was administered to all groups to eliminate potential dietary interference with systemic tyrosine levels and metabolomic outcomes. At the same time, we carefully monitored and recorded the daily food intake for each mouse cage. The average daily intake per mouse was approximately 2 g, and no siginificant differences were observed between the groups. Thus, it appears that dietary factors had minimal influence on the experimental results. Since dietary conditions were kept consistent across all groups, it is unlikely that variations in food intake affected the experimental data.

During DSS exposure, mice were treated once daily by gavage: the Low-dose and High-dose QYD groups received 6.295 and 12.59 g/kg QYD extract [12], respectively; the 5-ASA group received 200 mg/kg 5-aminosalicylic acid; the Control and DSS groups received 0.5% CMC-Na. The QYD extract was provided by the Affiliated Hospital of Nanjing University of Chinese Medicine, and dosages were guided by preliminary data.

For the *D. longicatena* pharmacodynamic study, mice were assigned to four groups: Control, DSS, Live *D. longicatena*, and Dead *D. longicatena*. The DSS, Live *D. longicatena*, and Dead *D. longicatena* groups received 3% DSS for 7 days to induce colitis, while the Control group received distilled water. During induction, mice in the Live and Dead *D. longicatena* groups were orally administered 2 × 10⁸ CFU/mouse daily [13]; the Control and DSS groups received 0.5% CMC-Na.

For tyrosine validation experiments, mice were divided into three groups: Control, DSS, and Tyrosine. The DSS and Tyrosine groups received 3% DSS for 7 days, while the Control group received distilled water. During this period, the Tyrosine group was given 100 mg/kg tyrosine daily by oral gavage; the Control and DSS groups received 0.5% CMC-Na. Body weight, stool consistency, and rectal bleeding were recorded daily, and the DAI was calculated based on weight loss, stool consistency, and bleeding (score 0–4 for each) (Table S1) [14].

### Methods for preparing inactivated *Dorea longicatena*

Harvesting and Washing: *D. longicatena* cultures were harvested during the logarithmic growth phase. The bacterial cells were pelleted by centrifugation at 4000 rpm for 10 min at 4 °C. The supernatant was carefully discarded, and the cell pellet was washed twice with sterile PBS using the same centrifugation parameters.

Inactivation and Validation: The final washed pellet was resuspended in sterile PBS and adjusted to 2 × 10⁸ colony-forming units per mouse. Complete inactivation was achieved by autoclaving the suspension at 121 °C for 15 min. The sterility of the inactivated preparation was confirmed by the absence of microbial growth following plating on appropriate agar media [15, 16].

Storage: The inactivated bacterial suspension was aliquoted and stored at 4 °C until further use.

### Biological sample acquisition

All mice were euthanized under inhalation anesthesia in accordance with institutional animal ethics guidelines. In this study, each experimental group contained six animals, from which six independent tissue samples were collected and analyzed. Blood was collected via orbital enucleation, and serum was obtained by centrifugation [17]. Subsequently, a midline abdominal incision was made in a biosafety cabinet. The skin was fixed with pins, and the entire intestinal tract was gently exteriorized using sterile forceps and positioned on the right side of the body. The colon was then excised to measure its length. Colon contents were collected with a sterile spoon, flash-frozen in liquid nitrogen, and stored at − 80 °C for microbiome analysis. A 1–2 cm segment of distal colon tissue adjacent to the rectum was dissected, opened longitudinally with sterile scissors, rinsed thoroughly with phosphate-buffered saline (PBS), and blotted dry on sterile filter paper. One portion of the tissue was fixed in 4% paraformaldehyde (Aifang Biotechnology Co., Ltd. Hunan, China), while the remaining sample was placed in a centrifuge tube and stored at − 80 °C for subsequent analysis.

### Histological analysis

Colon tissue samples were fixed in 4% paraformaldehyde overnight, dehydrated, and embedded in paraffin. Sections with a thickness of 4 μm were prepared and stained with hematoxylin and eosin (H&E) for histopathological examination. The severity of intestinal injury was assessed in a blinded manner using a standardized histological scoring system, with higher scores indicating greater tissue damage [18]. The experiment was independently repeated three times, and data analysis was based on the average of three independent measurements per sample. The images presented are representative of the results obtained. To ensure clarity and consistency in visual presentation, specific histopathological features of interest in the representative photomicrographs are annotated with colored arrows: red arrows indicate ulceration, and black arrows indicate cryptitis. This standardized annotation scheme is applied consistently across all relevant figures in the study.

### Quantitative real-time PCR analysis of tissues and *D. longicatena*

Total RNA was isolated from colon tissues using the FastPure Complex Tissue/Cell Total RNA Isolation Kit (Vazyme, RC113). RNA concentration and purity were determined with a DeNovix DS-11FX microvolume spectrophotometer. Subsequently, complementary DNA (cDNA) was synthesized from 0.5 μg of total RNA using the HiScript II Q RT SuperMix Kit (Vazyme). For bacterial analysis, genomic DNA was extracted from fecal samples with the SPINeasy DNA Kit (MP Biomedicals) following the manufacturer’s instructions. Gene expression was quantified by quantitative real-time PCR (qPCR) using the SYBR Green PCR Reagent Kit (Yeasen) on a QuantStudio 3 system (Thermo Fisher Scientific). The thermal cycling conditions were as follows: initial denaturation at 95 °C for 5 min, followed by 40 cycles of 95 °C for 10 s, 60 °C for 30 s, and 72 °C for 20 s.

To ensure accurate normalization, different endogenous controls were used for specific target amplicons. β-actin served as the reference gene for quantifying the mRNA expression levels of host inflammatory genes (e.g., *Tnf、Il1b).* For the quantification of bacterial load, the 16S rRNA gene (targeting the V3/V4 region) was amplified, and the total 16S rRNA gene copy number was used as the internal control for relative abundance calculation. Primer sequences are provided in Table S2.

Gene expression data were analyzed using the comparative ΔΔCt method for relative quantification. The cycle threshold (Ct) values were first normalized to the Ct value of the corresponding endogenous control gene to obtain the ΔCt value [ΔCt = Ct(target)–Ct(reference)]. The normalized ΔCt values for the experimental groups were then calibrated against the mean ΔCt of the control group to calculate the ΔΔCt value [ΔΔCt = ΔCt(sample)–Mean ΔCt(control)]. The final relative gene expression level or bacterial relative abundance was expressed as fold change calculated by the formula 2^(–ΔΔCt) [19].

In this study, to ensure result reliability, both biological and technical replicates were incorporated. Each experimental group consisted of six independent mouse colon tissue samples. For real-time quantitative PCR, each RNA sample was assayed in three technical replicate wells to minimize operational variability. Data are presented as the mean ± standard deviation of three biological replicates.

### Immunohistochemistry analysis

Paraffin-embedded colon tissue Sects. (4 μm) were deparaffinized and subjected to antigen retrieval using citrate buffer. After marking the tissue boundaries, endogenous peroxidase activity was quenched with 3% hydrogen peroxide. Sections were then blocked with 3% bovine serum albumin (BSA) at room temperature, washed, and incubated overnight at 4 °C with primary antibodies (anti-ZO-1, 1:100; anti-occludin, 1:100) (Proteintech). Following rewarming and washing, a rabbit anti-rabbit secondary antibody was applied at room temperature. Color development was achieved using 3,3′-diaminobenzidine (DAB) as the chromogenic substrate. Sections were counterstained with hematoxylin, differentiated in 1% hydrochloric acid–ethanol, dehydrated, cleared in graded ethanol and xylene, and mounted with neutral resin. Three random microscopic fields were selected from each section for analysis under a light microscope [20]. The expression levels of tight junction proteins ZO-1 and Occludin were quantified using a standardized immunoreactivity score (IRS) system that incorporates both staining intensity and the proportion of immunopositive cells. Staining intensity (A) was graded on a scale of 1–3 (1: light brown; 2: brown; 3: dark brown). The percentage of immunopositive cells (B) was scored on a scale from 1 to 10, corresponding to 10% increments (e.g., 1–10% = 1; 11–20% = 2; … > 91% = 10). The final IRS for each microscopic field was calculated as the product of A and B (A × B) [21, 22]. For each biological sample, five randomly selected high-power fields were evaluated. All slides were independently assessed by two pathologists blinded to the experimental conditions; any discrepancies in scoring were resolved by consensus. Three independent biological replicates were analyzed per experimental group. The average IRS for each sample was calculated from five fields, and group-level means were derived from the three biological replicates for statistical analysis and graphical representation. Data are presented as the mean of three independent measurements, with representative images shown.

### 16S rRNA gene sequencing

Frozen colonic contents were shipped on dry ice to LC-Bio Technologies (Hangzhou) Co., Ltd. (Hangzhou, China) for 16S rRNA gene sequencing. Genomic bacterial DNA extraction and quality assessment were performed by LC-Bio Technologies. The V3–V4 hypervariable regions of the bacterial 16S rRNA gene were amplified using forward primer: 341 F (5′-CCTACGGGNGGCWGCAG-3′) and reverse primer: 805 R (5′-GACTACHVGGGTATCTAATCC-3′). PCR amplification, DNA purification, and library quantification for paired-end sequencing were conducted by LC-Bio Technologies using an Agilent 2100 Bioanalyzer (Agilent, USA) and Illumina platform (Kapa Biosciences, Woburn, MA, USA). Raw sequencing data were demultiplexed, assembled, and filtered. Sequence denoising and length filtering were performed using the qiime dada2 denoise-paired command to invoke dada2. Amplicon sequence variant (ASV) feature tables and representative sequences were generated and used for α- and β-diversity analyses. Differential abundance analyses among groups were performed based on ASV abundance tables using Fisher’s exact test, Mann–Whitney U test, and Kruskal–Wallis test on the LC-Bio Cloud Platform (https://www.omicstudio.cn).

### Culture of *D. longicatena*

*D. longicatena* strain (DSM 13814) was obtained from Mingzhou Bio (Ningbo, Zhejiang, China) and cultured in CMC medium (TOPBIO, Shandong, China). The medium was supplemented with beef extract, 50 μg/mL vitamin K₁ (TOPBIO, Shandong, China), and 5 μg/mL hemin (TOPBIO, Shandong, China). Cultures were incubated statically at 37 °C for 48–72 h in an anaerobic workstation under an atmosphere of 10% CO₂, 10% H₂, and 80% N₂. Bacterial concentration was estimated based on optical density at 600 nm (OD₆₀₀) [23].

### Serum non-targeted metabolomics analysis

Serum samples (50 μL) were deproteinized with 200 μL of ice-cold methanol containing 1,2-^13^C-labeled myristic acid (12.5 μg/mL) as the internal standard. After vortex mixing and centrifugation, 100 μL of the supernatant was evaporated to dryness. The dried residue was subsequently methoximated with methoxyamine hydrochloride in pyridine (1.5 h, 30 °C), followed by trimethylsilylation with BSTFA (0.5 h, 37 °C). Quality control samples were prepared by pooling equal aliquots from all individual specimens [24].

GC–MS analysis was performed using a TRACE 1310/TSQ 8000 system equipped with a TG-5MS capillary column (30 m × 0.25 mm × 0.25 μm), with helium as the carrier gas at a flow rate of 1.2 mL/min. The temperature program started at 60 °C for 1 min, increased at 20 °C/min to 320 °C, and was held for 5 min. The injection volume was 1 μL with a 20:1 split ratio. Electron ionization (70 eV) was applied, and the ion source and transfer line temperatures were maintained at 280 °C and 250 °C, respectively. Mass spectra were acquired over an *m/z* range of 50–500.

GC–MS data were processed using MS-DIAL 4.9 for peak extraction, deconvolution, and alignment prior to metabolite identification against the NIST database. Multivariate statistical analyses, including PCA and OPLS-DA, were performed in SIMCA. Differential metabolites were selected based on VIP > 1 and FDR-adjusted *p* < 0.05. Metabolic pathway enrichment analysis was subsequently conducted using MetaboAnalyst 6.0.

### Targeted metabolomics for tyrosine

#### Sample pretreatment and derivatization of *D.longicatena* bacterial supernatant

Take 500 μL of blank culture medium and *D.longicatena* bacterial supernatant, respectively, add 500 μL of 0.005 mol/L NaOH aqueous solution, vortex for 2 min, let stand at 4 °C for 2 h, vortex again for 2 min, and centrifuge at 13,000 rpm for 20 min at 4 °C. The supernatant (500 μL) was mixed with 300 μL ultrapure water, 300 μL isopropanol, 200 μL pyridine, and 100 μL propyl chloroformate, followed by the addition of 300 μL hexane. After vortexing and centrifugation, the upper hexane layer (200 μL) was collected. The residue was re-extracted with 200 μL hexane, and both hexane layers were pooled, followed by addition of 10 mg anhydrous sodium sulfate. The supernatant was collected for GC–MS analysis [25].

#### Pre-treatment and derivatization of colonic content samples

A 30 mg colonic contents sample was mixed with 1 mL 0.005 mol/L NaOH. After homogenization and incubation at 4 °C for 2 h, the mixture was centrifuged (13,000 rpm, 10 min, 4 °C). A 500 μL aliquot of the supernatant was derivatized using the same procedure as that for *D. longicatena* bacterial supernatant samples [26].

#### Colon tissue samples pre-treatment and derivatization

Colon tissue (50 mg) was homogenized in 1000 μL 0.005 mol/L NaOH, then incubated at 4 °C for 2 h. After centrifugation (13,000 rpm, 10 min, 4 °C), a 500 μL aliquot of the supernatant was derivatized using the same procedure as for *D.longicatena* bacterial supernatant samples [27].

The analysis was conducted using an Agilent HB-5MS chromatographic column (30 m × 0.25 mm, 0.25 µm). A 1 µL sample was injected in split mode with a split ratio of 10:1, and the solvent delay time was set to 2.15 min. The injection port temperature was maintained at 250 °C. The temperature program was as follows: the initial temperature was held at 50 °C for 3.5 min; then, it was increased at a rate of 30 °C/min to 70 °C and held for 3.5 min; the temperature was increased at 30 °C/min to 85 °C and held for 5.722 min; next, it was increased at 40 °C/min to 130 °C and held for 5.722 min; then, the temperature was increased at 40 °C/min to 290 °C and held for 10.722 min; finally, the temperature was held at 290 °C for 2 min. Helium (He) was used as the carrier gas at a flow rate of 1.0 mL/min.

Mass spectrometry conditions: The ionization source was EI with an electron energy of + 70 eV. The ion source temperature was set to 230 °C, and the quadrupole temperature was 150 °C. The solvent delay time was 2.15 min. The collision energy for both ion pairs was 15 eV [28].

#### Label-free proteomics analysis

Protein digestion was performed using the single-pot, solid-phase-enhanced sample preparation (SP3) technology [29]. Briefly, 10 μg of protein was reduced with 10 mM DTT at 56 °C for 30 min, alkylated with 40 mM iodoacetamide (IAA) in the dark at room temperature for 30 min, and subsequently quenched with 20 mM DTT at room temperature for 10 min. The mixture was incubated with a 1:1 blend of hydrophilic and hydrophobic magnetic beads at a 10:1 bead-to-protein ratio (w/w) for 30 min at room temperature. After washing, the beads were digested with 0.5 μg trypsin in 50 μL of 50 mM NH₄HCO₃ at 37 °C overnight with shaking at 1500 rpm. Peptides were desalted using C18 StageTips [30], lyophilized, and reconstituted in 0.1% formic acid. They were loaded onto a 20-cm in-house packed column with C18 3 μm ReproSil particles (Dr. Maisch GmbH, r13.aq.). Separation was performed on an EASY-nLC 1200 system using solvent A (0.1% formic acid in water) and solvent B (80% acetonitrile, 0.1% formic acid). The gradient was as follows: 6%–32% B over 100 min, 32%–45% B over 6 min, 45%–100% B over 2 min, and 100% B for 12 min. Full-scan MS spectra (300–1500 m/z) were acquired with a maximum injection time of 100 ms and a resolution of 70,000. The AGC target was set to 3 × 10⁶. Data-independent acquisition (DIA) scans were performed at a resolution of 35,000 with 20 m/z isolation windows. HCD fragmentation used a normalized collision energy of 27%.

Raw files were analyzed with DIA-NN (v1.8.1) using a spectral library built from the UniProt mouse proteome (accessed November 2024) with "Deep learning-based spectra and RTs prediction" enabled. Precursor FDR was set to 1%; other parameters were default. Statistical analysis and visualization used R. Proteins quantified in ≥ 50% of samples in all three experimental groups were retained, with missing values imputed by KNN algorithm. P-values from t-tests were Benjamini–Hochberg-adjusted. Differentially expressed proteins met adjusted p < 0.05 for both Control vs. DSS and DSS vs. Tyr comparisons. PCA, Gene Ontology biological process and Kyoto Encyclopedia of Genes and Genomes (KEGG) pathway enrichment analyses were performed using the R packages.

### Statistical analysis

Statistical analyses were performed using Prism version 9.0 (GraphPad Software, CA, USA). Statistical differences were evaluated using an unpaired Student’s t-test or one-way analysis of variance (ANOVA) followed by Tukey’s post hoc test. The data were derived from multiple independent biological replicate experiments. It is explicitly noted that all statistical analyses were performed based on these biological replicates, not on technical replicates.

## Results

### QYD alleviated colitis symptoms in UC mice

**Fig. 1:**
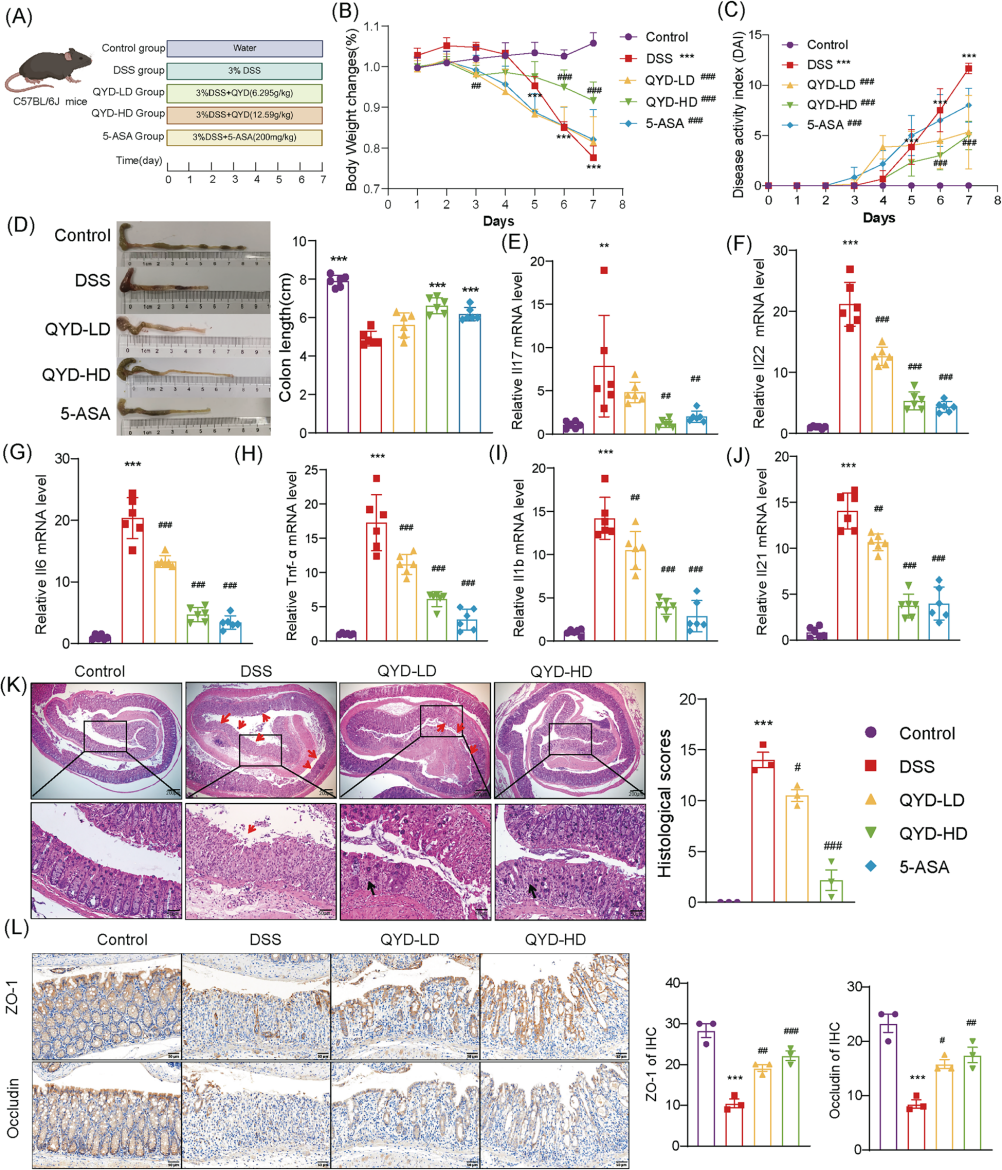
QYD ameliorates symptoms and colonic injury in DSS-induced UC mice. **A** Schematic diagram of the animal experiment workflow. **B** Body weight changes in each group (n = 6). **C** DAI scores change chart (n = 6). **D** Representative images and length comparisons of colonic tissue across groups. **E** IL-17 levels in colonic tissue (n = 6). **F** IL-22 levels in colon tissue (n = 6). **G** IL-6 levels in colon tissue (n = 6). **H** Tnf-α levels in colon tissue (n = 6). **I** IL-1β levels in colon tissue (n = 6). **J** IL-21 levels in colon tissue (n = 6). **K** Representative H&E staining images and scores of colon tissue (Red arrows indicate ulceration; black arrows indicate cryptitis) (n = 3). **L** IHC staining of ZO-1 and Occludin protein expression in colon sections (n = 3). *p < 0.05, **p < 0.01, ***p < 0.001 vs. Control group; ^#^*p* < 0.05, ^##^*p* < 0.01, ^###^*p* < 0.001 vs. DSS group. Data are expressed as Mean ± SD. Statistical comparisons between groups at each individual time point were performed using two-way ANOVA (B and C). Multiple comparisons were analyzed using one-way analysis of ANOVA (D-L)

In DSS-induced UC mice model, the ameliorative effects of QYD at various doses and mesalazine were assessed for 7 days (Fig. 1A). Mice treated with DSS exhibited significant weight loss by day 4 compared with the control group. Notably, administration of mesalazine and QYD attenuated weight loss in UC mice, with the high-dose QYD group showing the most pronounced effect. Moreover, weight recovery in the experimental group showed no statistically significant difference compared to the Control group, indicating a comparable restorative effect (Fig. 1B). In addition, DSS treatment induced severe diarrhea, hematochezia, and elevated DAI scores (Fig. 1C), whereas oral administration of mesalazine or QYD alleviated these symptoms. Specifically, DAI scores in the QYD-HD group exhibited a recovery, reaching levels that approached those observed in the Control group. Treatment with mesalazine or QYD, particularly at the higher dose, significantly prevented colon shortening and preserved mucosal integrity, leading to notable improvements in villous morphology and overall tissue structure (Fig. 1D, K). Histological analysis indicated that colonic mucosal injury in the QYD-HD group was comparable in degree to that observed in the Control group. DSS exposure significantly increased the expression of proinflammatory cytokines, whereas treatment with mesalazine or QYD reduced their expression. In the QYD-HD group, all measured cytokine levels approached or fell within the range observed in the Control group, demonstrating QYD efficacy in alleviating colonic inflammation (Fig. 1E–J). Compared with the DSS group, administration of QYD, significantly upregulated the expression of the tight junction proteins ZO-1 and Occludin. Moreover, the expression levels of these proteins in the QYD-HD group were found to be comparable to those in the Control group. (Fig. 1L). Collectively, these results demonstrate that QYD confers significant therapeutic benefits in DSS-induced UC.

### QYD increased the abundance of gut commensal *D. longicatena* in UC mice

**Fig. 2:**
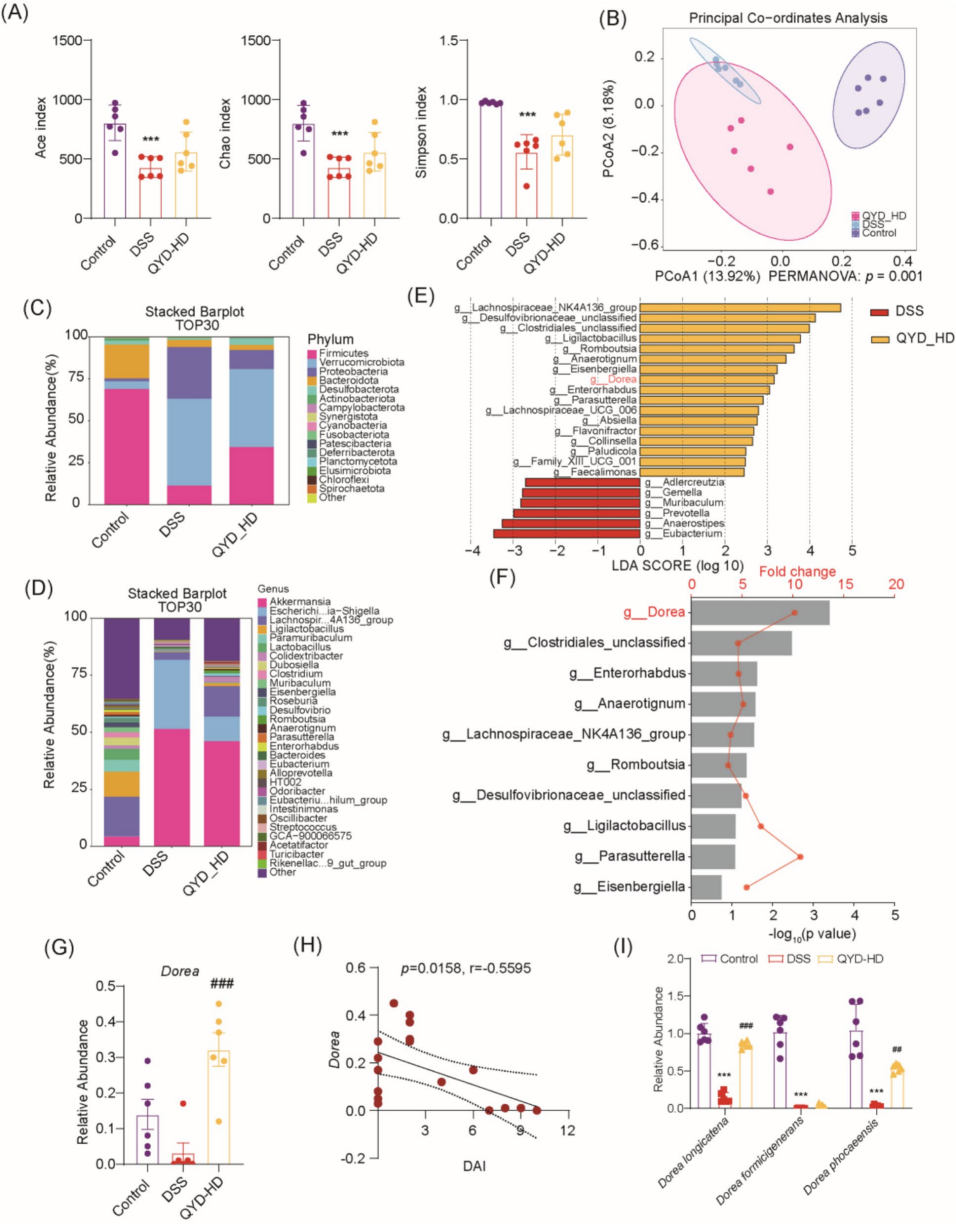
QYD alleviates gut dysbiosis in UC primarily by increasing *D. longicatena* abundance. **A** α-diversity indices: ACE, Chao1, and Simpson (n = 6). **B** PCoA analysis of gut microbiota in each group based on Jaccard distance (n = 6) (PERMANOVA: *p* = 0.001) PCoA plot showing sample distribution across groups. Group differences were tested using PERMANOVA. **C** Bar chart of relative species abundance at phylum level across groups (TOP 30). **D** Bar chart of relative species abundance at genus level across groups (TOP 30). **E** LEfSe analysis revealing significantly different bacterial communities between groups (LDA > 2) (n = 6). **F** Differential abundance analysis between DSS and QYD-HD groups (n = 6). **G** Abundance changes of *Dorea* genus before and after QYD administration (n = 6). **H** Correlation analysis between *Dorea* genus and UC pharmacodynamic indicator DAI score (n = 6). **I** The abundance of common *Dorea* species in colonic contents of UC mice (n = 6). *p < 0.05, **p < 0.01, ***p < 0.001 vs. Control group; ^#^*p* < 0.05, ^##^*p* < 0.01, ^###^*p* < 0.001 vs. DSS group. Multiple comparisons were performed using one-way ANOVA

To identify key microbiome mediating the therapeutic effects of QYD in UC, 16S rRNA sequencing was utilized. Comprehensive α-diversity analysis based on the ACE, Chao1, and Simpson indices revealed significant differences between the Control and DSS groups. However, no significant difference in α-diversity was observed between the DSS and QYD-treated groups (Fig. 2A). β-Diversity analysis showed distinct clustering of microbial communities in the Control and DSS groups, while QYD treatment shifted the overall microbial composition toward that of the Control group (Fig. 2B). At the phylum level, Bacteroidetes abundance exhibited a declining trend following DSS exposure, which was partially restored by QYD treatment (Fig. 2C). Genus level analysis indicated that QYD increased the abundance of *Lachnospiraceae_NK4A136_group*, *Ligilactobacillus*, *Paramuribaculum*, *Lactobacillus*, *Colidextribacter*, *Clostridium*, *Anaerotignum* and *Dorea*, while decreasing the abundance of *Akkermansia*, *Escherichia-Shigella*, *Eubacterium*, *Odoribacter* and *Intestinimonas* (Fig. 2D).

LEfSe analysis further confirmed the beneficial bacteria enriched after QYD administration, including *Lachnospiraceae_NK4A136_group*, *Ligilactobacillus*, *Romboutsia*, *Anaerotignum*, *Eisenbergiella*, and *Dorea* (Fig. 2E, Figure S1). To identify genera that were significantly increased after QYD treatment, differential abundance analysis was performed based on LEfSe results. *Dorea* exhibited the most significant inter-group differences and showed a significant negative correlation with DAI scores (Fig. 2F–H). We identified 3 species (*D. longicatena*, *D. formicigenerans*, *D. phocaeensis*) among the genus *Dorea*. *D. longicatena* was significantly increased in the fecal samples after QYD treatment (Fig. 2I). Collectively, these findings demonstrate that QYD ameliorates DSS-induced gut dysbiosis, especially by promoting the enrichment of *D. longicatena*, which may contribute to UC remission.

### Oral administration of live *D. longicatena* alleviated colitis symptoms in UC mice

**Fig. 3:**
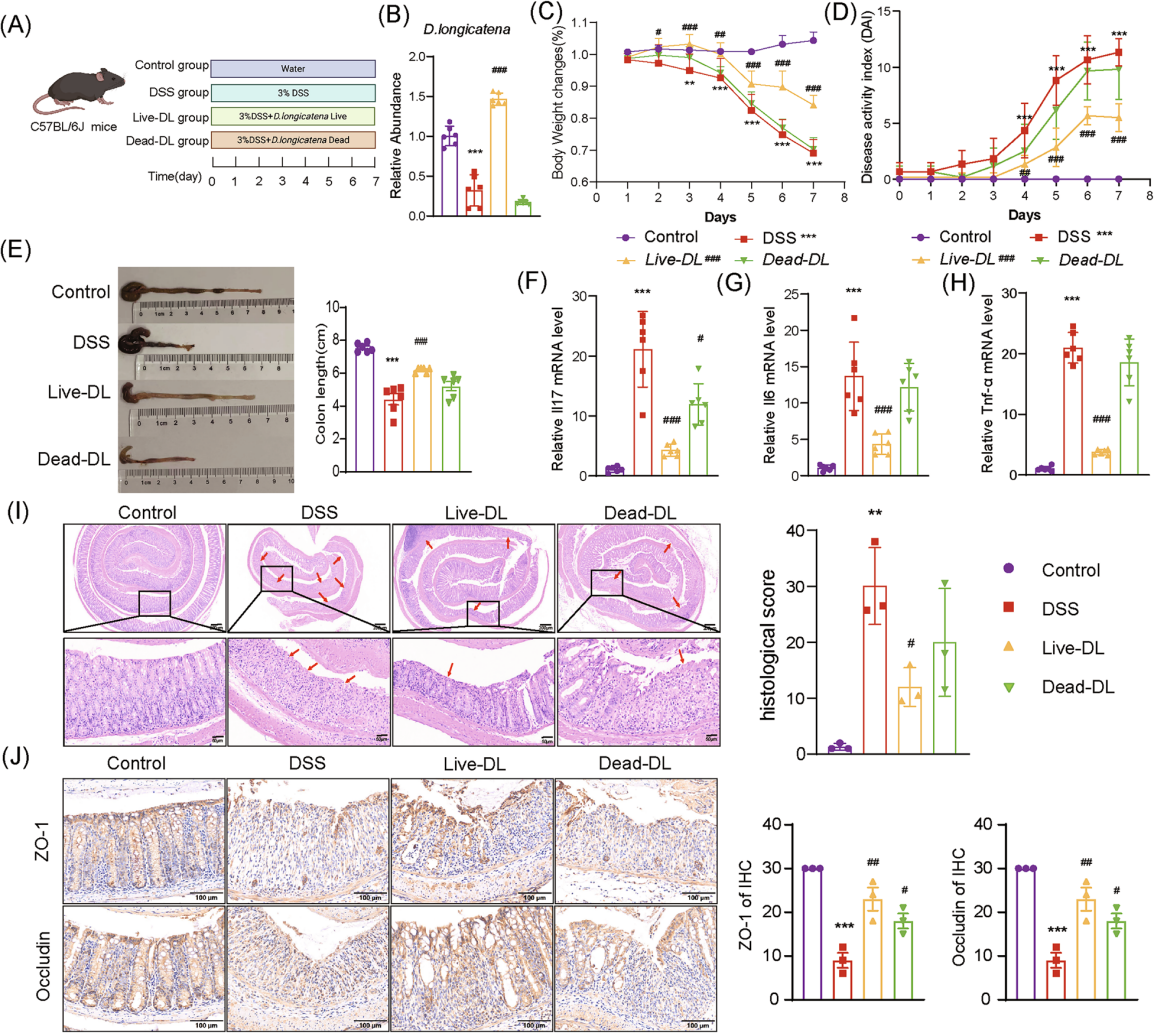
*D. longicatena* ameliorates symptoms and colonic damage in DSS-induced colitis mice. **A** Schematic diagram of the animal experiment workflow. **B** Changes in *D. longicatena* bacterial abundance in UC mice across groups (n = 6). **C** Body weight changes in mice across groups (n = 6). **D** DAI scores change chart (n = 6). **E** Representative images and colon lengths for comparison (n = 6). **F** Levels of IL-17 inflammatory factor in colonic tissue (n = 6). **G** Levels of IL-6 inflammatory factor in colonic tissue (n = 6). **H** Levels of Tnf-α inflammatory factor in colonic tissue (n = 6). **I** Representative H&E staining images and scores of colonic tissues (n = 3). **J** IHC staining of ZO-1 and Occludin protein expression in colonic sections and scoring (Red arrows indicate ulceration; black arrows indicate cryptitis) (n = 3). Data are expressed as mean ± SD. *p < 0.05, **p < 0.01, ***p < 0.001 vs. Control group; ^#^*p* < 0.05, ^##^*p* < 0.01, ^###^*p* < 0.001 vs. DSS group. Statistical comparisons between groups at each individual time point were performed using two-way ANOVA (**C** and **D**). Multiple comparisons were analyzed using one-way analysis of ANOVA (**B** and **E**–**J**)

Evaluate the therapeutic potential of *D. longicatena* in UC mice (Fig. 3A). The results showed that the abundance of live *D. longicatena* significantly increased in UC mice, while the abundance of mice receiving dead *D. longicatena* was comparable to the DSS group (Fig. 3B). These findings confirm that *D. longicatena* can effectively colonize. Compared to the Control group, mice in the DSS group exhibited significant weight loss. However, treatment with live *D. longicaten*a significantly attenuated this loss, restoring body weight to a level indistinguishable from that of the Control group (Fig. 3C). In addition, live *D. longicatena* intervention significantly alleviated DSS-induced diarrhea, hematochezia, and elevated DAI scores, restoring the DAI to a level comparable to that of the Control group. In contrast, heat-killed *D. longicatena* had no appreciable effect (Fig. 3D). Notably, live *D. longicatena* substantially mitigated colon shortening and restored mucosal integrity. Histological evaluation revealed that colonic structural damage in the treatment group had recovered to a level comparable to that of the Control group. In contrast, the dead *D. longicatena* group showed changes similar to those in the DSS group (Fig. 3E, I). RT-qPCR analysis revealed upregulation of pro-inflammatory cytokines in the DSS group. Treatment with live *D. longicatena* significantly reduced these levels, which subsequently gradually returned to the range observed in the Control group, indicating potent suppression of colonic inflammation (Fig. 3F–H). Furthermore, compared to the DSS group, treatment with live *D. longicatena* significantly upregulated the expression of the tight junction proteins ZO-1 and Occludin, which restored their levels to those of the Control group and repaired the gut barrier. (Fig. 3J). Overall, these findings demonstrate that *D. longicatena* is beneficial and protective against UC.

### QYD modulated serum metabolic profiles in UC mice

**Fig. 4:**
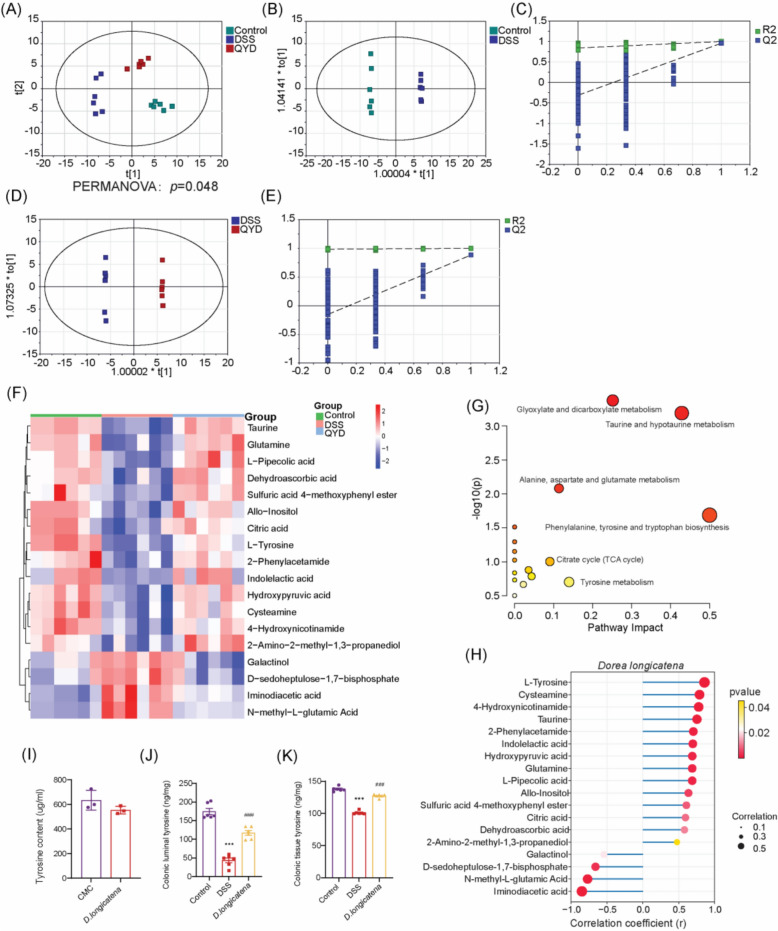
Metabolomics analysis exploring the metabolic mechanisms of QYD in UC mice (n = 6). **A** PCA score plot comparing Control, DSS, and QYD-HD groups. (PERMANOVA: *p* = 0.048) **B** OPLS-DA score plot comparing Control vs. DSS groups. **C** 200-replacement test plot of the OPLS-DA model for Control vs. DSS groups. **D** OPLS-DA score plot comparing DSS vs. QYD-HD groups. **E** 200-replacement test plot of the OPLS-DA model for DSS vs. QYD-HD groups. **F** Heatmap analysis of differential metabolites among Control, DSS, and QYD-HD groups. **G** Bubble chart of KEGG pathway enrichment analysis for differentially metabolized compounds among the three groups. **H** Correlation analysis between differential metabolites and the gut bacterium *D. longicatena*. PCA plot showing sample distribution across groups. Group differences were tested using SIMCA. **I** Supernatant tyrosine levels in *D. longicatena* culture measured by GC–MS (ng/mL) (n = 3). **J** Colonic luminal tyrosine levels in DSS-induced colitis mice supplemented with *D. longicatena* (ng/mg) (n = 6). **K** Colonic tissue tyrosine levels in DSS-induced colitis mice supplemented with *D. longicatena* (ng/mg) (n = 6). *p < 0.05, **p < 0.01, ***p < 0.001 vs. Control group; ^#^*p* < 0.05, ^##^*p* < 0.01, ^###^*p* < 0.001 vs. DSS group. Multiple comparisons were performed using one-way ANOVA

To compare metabolic profiles among groups, PCA was used to assess intergroup variation. The Control and DSS groups clustered separately with no overlap. Following QYD treatment, the metabolic profile of mouse serum shifted toward that of the Control group (Fig. 4A). The supervised classification model OPLS-DA was subsequently used for further analysis. Clear separation was observed between the Control and DSS groups, as well as between the DSS and QYD-HD groups (Fig. 4B, D). To prevent overfitting of the original model, permutation tests were performed with 200 iterations. The R^2^ and Q^2^ values of the original OPLS-DA model were substantially higher than the corresponding values of the permuted models, indicating that the model is not overfitted (Fig. 4C, E).

VIP represents the variable weight value of the OPLS-DA model and indicates the contribution of a variable to the variance explanation. Based on the OPLS-DA results, the metabolites were selected based on VIP scores > 1 and FDR adjusted *p*-values (FDR-*p*) < 0.05 (Table S3-4). DSS intervention resulted in significant changes in 53 metabolites compared to the control group, correlated with glyoxylate and dicarboxylate metabolism, taurine and hypotaurine metabolism, phenylalanine, tyrosine and tryptophan biosynthesis et al. (Figure S2A, C). Notably, QYD treatment regulated 26 of the HFD-induced metabolite changes, involving pathways such as beta-Alaine metabolism, taurine and hypotaurine metabolism, phenylalanine, tyrosine and tryptophan biosynthesis et al. (Figure S2B, D). By cross-comparisons of different groups, a total of 18 differential metabolites were identified, including L-tyrosine, cysteamine, allo-inositol, dehydroascorbic acid, citric acid et al. The altered metabolic pathways showed for glyoxylate and dicarboxylate metabolism, phenylalanine, tyrosine and tryptophan biosynthesis, taurine and hypotaurine metabolism et al. (Fig. 4F-G).

Correlation analysis between the shared differential metabolites and the gut bacterium *D. longicatena* revealed a significant association between tyrosine and *D. longicatena* (Fig. 4H), providing mechanistic support for subsequent investigations. To investigate the direct mechanism by which *D. longicatena* regulates intestinal tyrosine levels, we performed targeted metabolomic analysis on its bacterial supernatant. The results showed that, under pure culture conditions, there was no significant difference in tyrosine levels between the blank culture medium group and the *D. longicatena* supernatant group, with the measured values being nearly equivalent (Fig. 4I). This indicates that *D. longicatena* does not directly produce or secrete tyrosine. This finding rules out the possibility that it increases intestinal tyrosine levels through a simple biosynthetic pathway, suggesting that its regulatory effect may depend on host metabolism or complex interactions within the gut microbial ecosystem.

Based on this finding, we conducted *D. longicatena* experiments on DSS-treated mice and performed targeted tyrosine assays on mouse colonic contents and colonic samples. The results demonstrated that in the colon lumina contents, the tyrosine levels in the DSS group were significantly lower than those in the Control group, confirming the relationship between colitis and tyrosine depletion. More importantly, supplementation with *D. longicatena* alone significantly reversed this deficiency, restoring the lumina tyrosine levels to those comparable to the Control group, and significantly higher than the DSS group (Fig. 4J). Similarly, analysis of the colonic tissue contents revealed that the tyrosine levels in the DSS group were significantly lower than those in the Control group. Administration of *D. longicatena* significantly increased the tissue tyrosine levels in the DSS group, restoring them to levels comparable to the Control group (Fig. 4K). These findings further support the potential role of *D. longicatena* in alleviating ulcerative colitis through modulation of tyrosine metabolism in vivo, suggesting that QYD may exert its therapeutic effects via this mechanism.

### Exogenous tyrosine supplementation alleviated colitis symptoms in UC mice

**Fig. 5:**
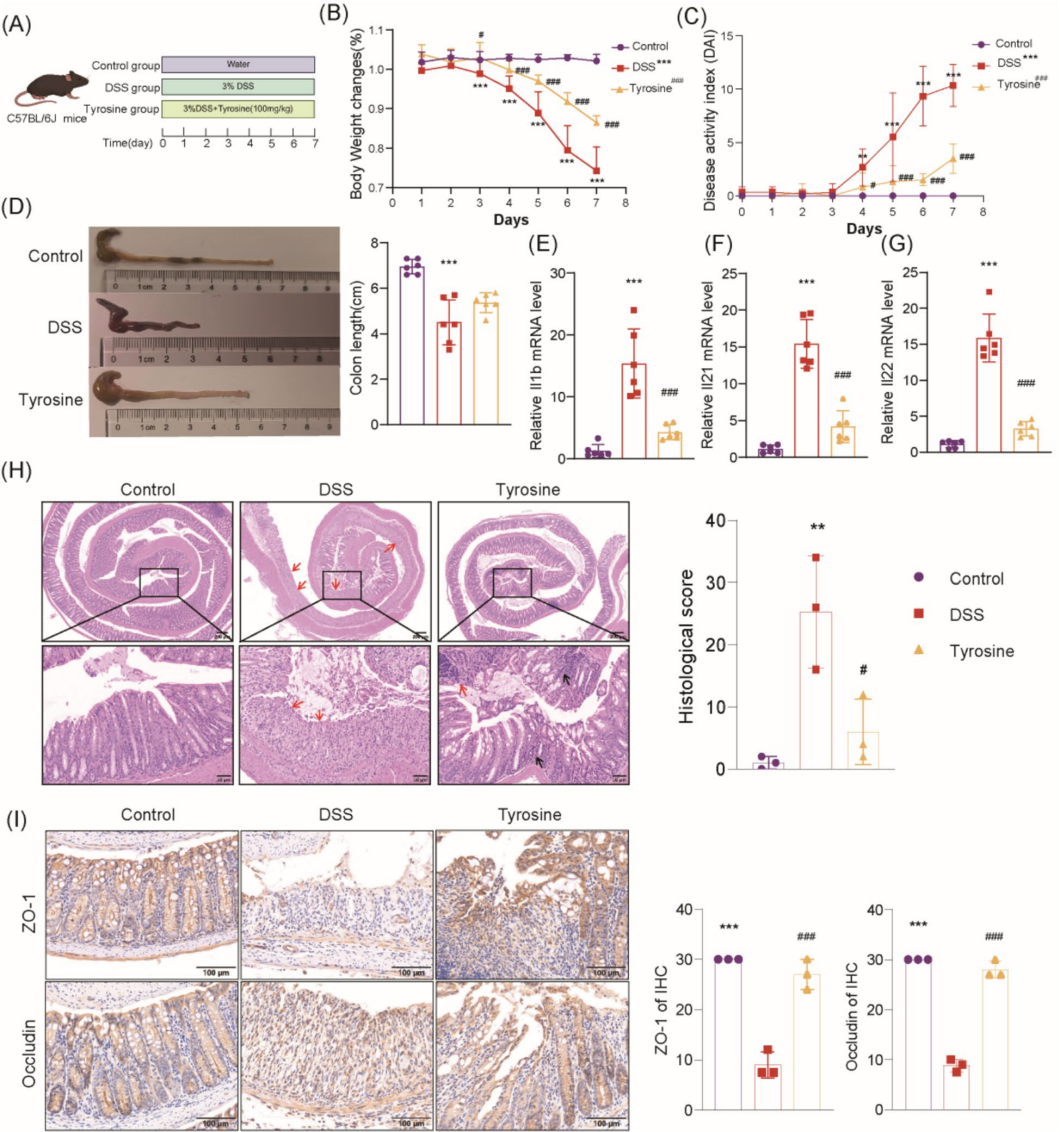
Tyrosine effectively alleviates symptoms and colonic damage in DSS-induced UC mice. **A** Schematic diagram of the animal experiment workflow. **B** Body weight changes in each group of mice (n = 6). **C** DAI scores change chart (n = 6). **D** Representative images and colon lengths for comparison (n = 6). **E** Levels of IL-1β inflammatory factor in colonic tissue (n = 6). **F** Levels of IL-21 inflammatory factor in colon tissue (n = 6). **G** Levels of IL-22 inflammatory factor in colon tissue (n = 6). **H** Representative H&E staining images and scores of colon tissue (Red arrows indicate ulceration; black arrows indicate cryptitis) (n = 3). **I** IHC staining of ZO-1 and Occludin protein expression in colon sections and scoring (n = 3). Data are expressed as mean ± SD. *p < 0.05, **p < 0.01, ***p < 0.001 vs. Control group; ^#^*p* < 0.05, ^##^*p* < 0.01, ^###^*p* < 0.001 vs. DSS group. Statistical comparisons between groups at each individual time point were performed using two-way ANOVA (**B** and **C**). Multiple comparisons were analyzed using one-way analysis of ANOVA (**D**–**I**)

To investigate the therapeutic role of tyrosine in UC, an acute UC mouse model was induced using DSS (Fig. 5A). Mice in the DSS group showed significantly reduced colon length, body weight loss, and elevated DAI scores compared with controls (Fig. 5B–D). In contrast, administration of tyrosine at 100 mg/kg restored colon length, mitigated weight loss, and lowered DAI scores to comparable levels. The relevant parameter values in the treatment group approached those observed in the Control group, showing a trend toward normalization (Fig. 5B–D). Tyrosine treatment also downregulated the DSS-induced expression of pro-inflammatory cytokines, including IL-21, IL-1β, and IL-22, and their expression levels showed a recovery trend toward those observed in the Control group, indicating its anti-inflammatory effects (Fig. 5E–G). Histological analysis by H&E staining revealed substantial ulcerative damage and increased cellular infiltration in DSS-treated mice, whereas these pathological alterations were reversed following tyrosine administration (Fig. 5H). In addition, compared to the DSS group, tyrosine enhanced the expression of tight junction proteins, such as Occludin and ZO-1 (Fig. 5I). The findings suggest a potential protective effect on colonic mucosal and epithelial integrity. Collectively, these results demonstrate that tyrosine effectively attenuates colonic inflammation and reinforces the intestinal barrier, supporting its therapeutic potential for UC.

### Proteomics analysis of colon tissue in UC mice treated with tyrosine

**Fig. 6:**
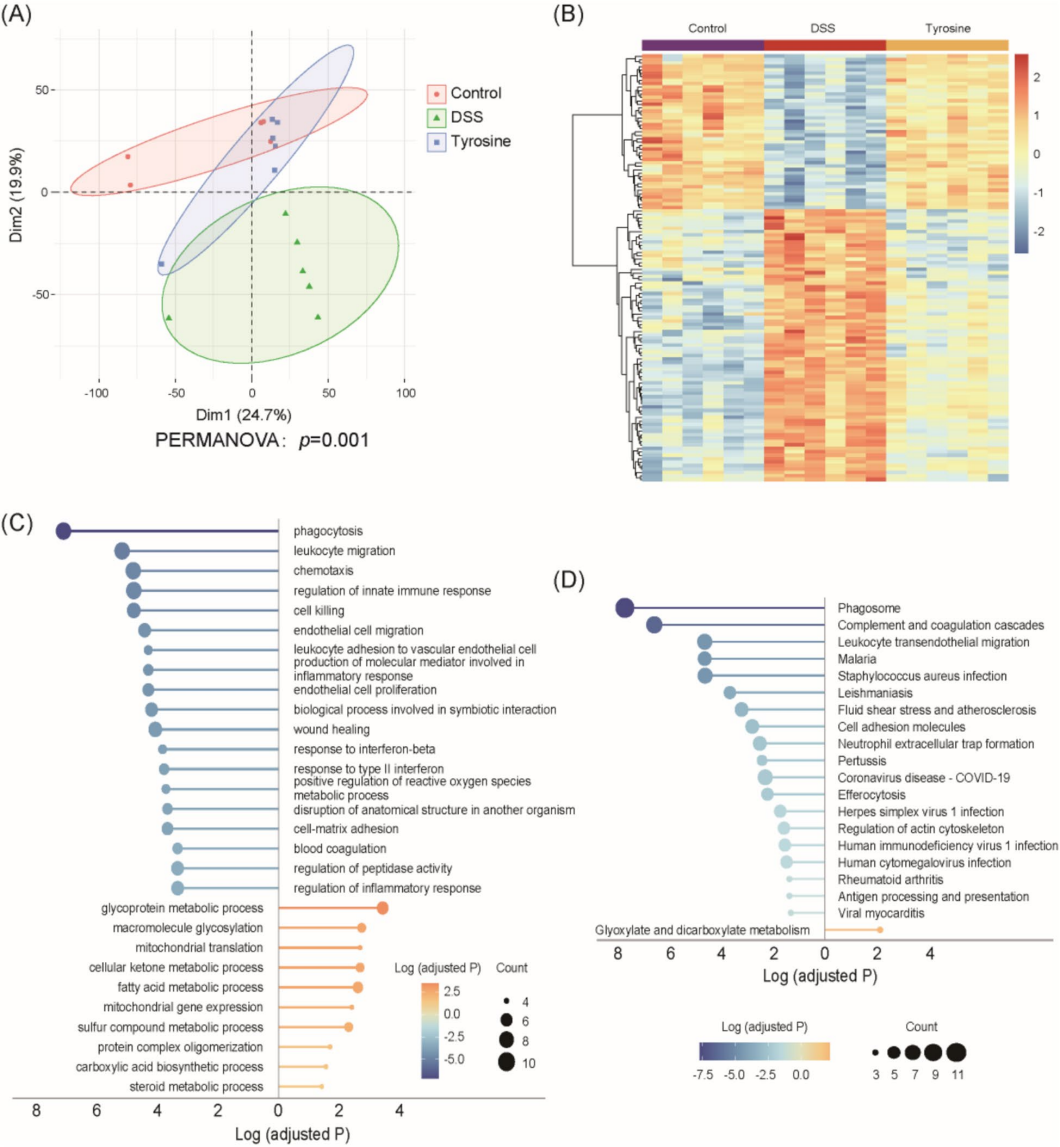
Proteomics analysis of DSS-treated mice treated with tyrosine. **A** PCA analysis plots for Control, DSS, and Tyr groups (PERMANOVA: *p* = 0.001) (n = 6). **B** Clustered Heatmap of Differentially Expressed Proteins Among Control, DSS, and Tyr Groups (n = 6). **C** Lollipop plot of GO enrichment analysis of DEPs. Gene Ontology terms significantly enriched among DEPs across Control, DSS, and Tyr groups are displayed. Blue circles represent GO terms downregulated in Tyr compared to DSS; yellow circles represent GO terms upregulated in Tyr compared to DSS. **D** Lollipop plot of KEGG enrichment analysis of DEPs. Kyoto Encyclopedia of Genes and Genomes pathways significantly enriched among DEPs across Control, DSS, and Tyr groups are shown. Blue circles indicate pathways downregulated in Tyr relative to DSS; yellow circles indicate pathways upregulated in Tyr relative to DSS

To investigate the potential targets and molecular mechanisms of tyrosine in UC, we applied proteomic profiling of colonic tissues from mice. In total, 8,420 proteins were identified, among which 6,302 proteins were quantified in at least 50% of the biological replicates across all three experimental groups. PCA demonstrated clear separation among the Control, DSS, and Tyr groups. (Fig. 6A). A comparative analysis of differential protein expression was carried out for the Control versus DSS groups and the DSS versus Tyrosine groups. Across these comparisons, 124 proteins were identified as differentially expressed proteins (DEPs), all satisfying the significance threshold (adjusted *p* < 0.05 in both the Control vs. DSS and DSS vs. Tyrosine comparisons) (Fig. 6B, Table S5).

To further elucidate the biological processes affected by tyrosine treatment, Gene Ontology (GO) functional and KEGG pathway enrichment analyses were conducted for the DEPs. The results indicate that tyrosine primarily perturbs multiple signaling pathways related to inflammation, immune response, and mucosal vascular function. GO biological process analysis showed that the down-regulated proteins were significantly enriched in processes related to inflammation and immune responses, including phagocytosis, leukocyte migration, chemotaxis, regulation of innate immune responses, cell killing, production of molecular mediators involved in inflammation, response to interferon-beta, response to type II interferon, positive regulation of reactive oxygen species metabolic process and regulation of inflammatory response (Fig. 6C). Furthermore, tyrosine significantly downregulated proteins involved in vascular endothelial structure and function, which were notably enriched in the biological processes of endothelial cell migration, endothelial cell proliferation, and leukocyte adhesion to vascular endothelial cells. The up-regulated proteins were predominantly implicated in metabolic and biosynthetic processes, such as glycoprotein metabolism, macromolecule glycosylation, mitochondrial translation, fatty acid metabolism, carboxylic acid biosynthesis, and steroid metabolism. These proteomic changes were consistent with corresponding alterations in key metabolites identified through metabolomic profiling after QYD treatment, including taurine, L-valine, citric acid, L-tyrosine and dehydroascorbic acid. Specifically, the enrichment of mitochondrial translation and fatty acid metabolic pathways corresponded with increased levels of citric acid, a pivotal metabolite in mitochondrial energy metabolism. Similarly, the upregulation of glycoprotein metabolic processes was associated with changes in taurine and L-tyrosine, both of which participate in protein modification and antioxidant responses. These findings suggest that tyrosine exerts anti-inflammatory and mucosal barrier-protective effects in UC by suppressing inflammation, modulating immune responses, inhibiting angiogenesis, and promoting mitochondrial and fatty acid metabolic homeostasis. Beyond alleviating UC by suppressing inflammatory responses, tyrosine may also contribute to mucosal barrier protection via the regulation of vascular endothelial function, while also promoting metabolic adaptation. KEGG pathway enrichment analysis revealed that tyrosine treatment significantly upregulated the glyoxylate and dicarboxylate metabolism pathway, while downregulating the phagosome, leukocyte transendothelial migration, and neutrophil extracellular trap formation pathways (Fig. 6D). Consistently, these findings indicate that tyrosine ameliorates UC symptoms primarily by suppressing inflammatory signaling pathways.

In summary, tyrosine alleviates inflammatory responses by suppressing the migration and phagocytosis of inflammatory cells as well as the production of inflammatory factors. Furthermore, it contributes to intestinal damage repair by regulating innate immune responses, modulating interferon signaling, and inhibition of angiogenesis. These findings provide theoretical support for elucidating the molecular mechanisms underlying the therapeutic potential of tyrosine in UC.

## Discussion

This study investigated the therapeutic effects of QYD in a mouse model of DSS-induced ulcerative colitis. The DSS-treated mice successfully recapitulated the key pathological features of human ulcerative colitis, including weight loss, diarrhea, rectal bleeding, and colon shortening [31]. QYD treatment significantly alleviated these symptoms, with the high-dose group showing efficacy comparable to that of 5-aminosalicylic acid, a first-line clinical drug. Moreover, QYD markedly upregulated the expression of tight junction proteins ZO-1 and Occludin in colonic tissues, further supporting the view that restoring intestinal barrier integrity is a critical therapeutic strategy for UC [32]. Intestinal barrier dysfunction is considered a central pathological event in UC [33], and multi-component herbal formulations may offer advantages in repairing barrier injury through multi-target synergistic mechanisms. This study provides robust experimental evidence for the therapeutic potential of QYD in UC, demonstrating its dual benefits in alleviating disease symptoms and restoring intestinal barrier function.

Gut microbiota dysbiosis is a hallmark of UC pathogenesis, characterized by reduced beneficial bacteria and increased opportunistic pathogens [34]. 16S rRNA sequencing revealed a significant decrease in the abundance of the genus *Dorea* in the intestines of DSS-induced mice, consistent with previous reports in both ulcerative colitis patients and animal models [35]. In recent years, *Dorea*, particularly its representative species *D. longicatena*, has gained increasing attention for its potential probiotic properties. Although some studies have suggested that *Dorea* may contribute to butyrate production and immune modulation [36], direct pharmacological evidence for its specific role in UC and the regulatory effects of traditional Chinese medicine on this bacterium remain limited. In this study, QYD treatment not only reversed DSS-induced depletion of *Dorea* but also, through in vivo supplementation with live *D. longicatena*, demonstrated significant protective effects against colitis and inflammation, along with improved intestinal barrier function. These findings suggest that QYD exerts its therapeutic effects by remodeling the gut microbiota, at least in part through restoring the abundance of *D. longicatena*.

Following the identification of *D. longicatena* as the most substantially altered taxon via 16S rRNA sequencing. RT-qPCR validation and in vivo supplementation experiments collectively offered support for the potential biological relevance of *D. longicatena* as a mediator of the therapeutic effects of QYD. Although *D. longicatena* emerged as the most prominent taxon in our multi-omics screening, we acknowledge that other microbial species may also contribute to the observed physiological effects. Future studies incorporating longitudinal monitoring of multiple candidate taxa, combined with multi-omics analyses, will help elucidate whether *D. longicatena* acts independently or as part of a broader microbial network reorganization.

Metabolic disturbances are common in UC [37], with amino acid metabolism abnormalities being particularly prominent [38]. This study identified tyrosine as a core metabolite regulated by QYD, which significantly enhances the tyrosine metabolic pathway and elevates its levels. Tyrosine not only serves as a substrate for protein synthesis but also acts as a precursor for neurotransmitters and immunomodulatory molecules essential for maintaining intestinal immune homeostasis [39–41]. Exogenous tyrosine supplementation has demonstrated clear therapeutic effects in UC mouse models. QYD intervention restored the abundance of *D. longicatena*, a bacterium that may influence host tyrosine levels through its metabolic activities. In vitro experiments showed that *D. longicatena* does not directly produce tyrosine; however, in vivo studies confirmed that this bacterium elevates tyrosine levels in colitic mice, suggesting that its regulatory effects depend on host or gut microenvironmental involvement.

Integrating in vivo and in vitro evidence, *D. longicatena* likely regulates host tyrosine levels by modulating the activity of key enzymes involved in tyrosine metabolism in the host and/or microbiota, such as tyrosine aminotransferase [42] and tyrosine hydroxylase [43]. Thus, *D. longicatena* may serve not only as a marker of tyrosine metabolism but also as a functional participant in this process. This suggests that, compared to medication alone, adjusting the dietary intake of tyrosine and its metabolic precursors, including deep-sea fish, or employing other nutritional interventions, may confer additional metabolic benefits for patients with UC.

Through quantitative proteomic profiling, we discovered that tyrosine alleviates UC via three primary pathways: suppression of inflammation, modulation of immune responses, and inhibition of angiogenesis. UC pathogenesis involves inflammation, immune dysregulation, barrier defects, and microbiome disturbances. Under tyrosine treatment, key proteins involved in inflammatory signaling were altered. For instance, S100A9, a clinical biomarker of neutrophil infiltration and disease activity in UC, was downregulated [44]. Additionally, Alox5ap, a critical adaptor in leukotriene biosynthesis that mediates lipid-inflammatory pathways central to UC pathogenesis, was reduced [45]. The leukocyte transendothelial migration-related proteins Icam1 and Vcam1 were also downregulated [46]. Tyrosine further modulated immune responses by downregulating STING1 [47], an endoplasmic reticulum protein essential for type I interferon and pro-inflammatory cytokine production, as well as the guanylate-binding proteins GBP2 [48] and GBP5 [49], which activate NLRP3 inflammasome assembly and function in both innate immunity and inflammation. Angiogenesis and vascular remodeling play key roles in UC progression, where hypoxia, immune cell infiltration, and pro-inflammatory cytokines collectively drive excessive vessel formation, thereby sustaining inflammation and mucosal barrier injury [50]. Our study showed that tyrosine significantly downregulated multiple pro-angiogenic and remodeling-related proteins, including LRG1 [51], PECAM-1 [52], and C5aR1 [53].

In summary, this study employed an integrated multi-omics approach to systematically elucidate the multi-level mechanisms underlying the therapeutic effects of QYD in UC. In addition to confirming its promising preclinical efficacy, we established a direct link between QYD treatment and the restoration of *D. longicatena*, a key gut commensal, accompanied by tyrosine. Tyrosine not only serves as a precursor for bioactive substances involved in neuroimmune regulation and intestinal barrier maintenance, but is also identified in this study as a core metabolite mediating the therapeutic effects of QYD. These findings outline a "microbiota–metabolite–host" regulatory axis, offering a novel perspective on the mechanisms of herbal formulations and providing scientific evidence for the concept of "medicine and food homology.

## Conclusions

In summary, QYD effectively alleviates DSS-induced UC by suppressing colonic inflammation and restoring intestinal barrier integrity. The mechanism underlying its therapeutic effects involves the upregulation of *D. longicatena* abundance in the gut and modulation of tyrosine metabolism. This study offers new insights into the molecular mechanisms of QYD in the treatment of UC, providing a foundation for its development as a novel therapeutic agent for this condition.

## Supplementary Information

Supplementary Material 1.

## Data Availability

The datasets supporting the conclusions of this article are available in multiple publicly accessible repositories. The 16S rRNA sequencing data are available in the NCBI Sequence Read Archive repository, BioProject accession PRJNA1425801 ( https://www.ncbi.nlm.nih.gov/). The metabolomics data are available in the OMIX database repository, accession PRJCA058641 ( https://ngdc.cncb.ac.cn/omix/). The proteomics data are available in the iProX database repository, accession IPX0015858000 ( https://www.iprox.cn/).

## Abbreviations

QYD: Qinlian Yuyang Decotion
UC: Ulcerative colitis
GQD: Gegen Qinlian Decoction
DSS: Dextran sulfate sodium
DAI: Disease activity index
5-ASA: 5-Aminosalicylic acid
H&E: Hematoxylin and eosin
IHC: Immunohistochemistry
IRS: Immunoreactivity score
PCoA: Principal coordinates analysis
LDA: Linear discriminant analysis
LEfSe: Linear discriminant analysis effect size
KEGG: Kyoto Encyclopedia of Genes and Genomes
GO: Gene ontology
PCA: Principal component analysis
OPLS-DA: Orthogonal Partial Least Squares Discriminant Analysis
Vip: Variable importance in projection
FDR: False discovery rate
