# Supplementary material for: Exploring the mechanism of Qinlian Yuyang Decoction in the treatment of ulcerative colitis based on multi-omics technology
Source: Chin Med. 2026 Mar 20;21:100. doi: 10.1186/s13020-026-01380-6 (PMC13003747; doi:10.1186/s13020-026-01380-6)
Supplement: Supplementary file 1 — Supplementary Material 1. [file 13020_2026_1380_MOESM1_ESM.docx]

**Additional file 1**

**Exploring the Mechanism of Qinlian Yuyang Decoction in the Treatment of Ulcerative Colitis Based on Multi-Omics Technology**

Ya-ting Cao^1,2+^, Xin Huang^1,2+^, Cheng-li Yu^3+^, Jing Wang^4+^, Xue Han^1,2^, Chen-wen Wang^5^, Zi-chen Luo^6^, Wei-chen Xu^6^, Jin-jun Shan^6^, Yong-ming Li^7^, Kang Ding^8*^, Ye Zhang^5*^, Ai-ling Yin^1,2*^

^1^Medical Experimental Centre, Central Laboratory, Nanjing Hospital of Chinese Medicine Affiliated to Nanjing University of Chinese Medicine, Nanjing, 210022, Jiangsu, China

^2^ Department of Biobank, Nanjing Hospital of Chinese Medicine Affiliated to Nanjing University of Chinese Medicine, Nanjing, 210022, Jiangsu, China

^3^ Jiangsu Key Laboratory for Functional Substances of Chinese Medicine, School of Pharmacy, Nanjing University of Chinese Medicine, Nanjing, 210023, Jiangsu, China

^4^ State Key Laboratory of Oral Drug Delivery Systems of Chinese Materia Medica, Affiliated Hospital of Integrated Traditional Chinese and Western Medicine, Nanjing University of Chinese Medicine, Nanjing, 210028, Jiangsu, China

^5^ State Key Laboratory of Natural Medicines, School of Traditional Chinese Pharmacy, China Pharmaceutical University, Nanjing, 211198, Jiangsu, China

^6^ Institute of Pediatrics, Jiangsu Key Laboratory of Pediatric Respiratory Disease, Medical Metabolomics Center, Nanjing University of Chinese Medicine, Nanjing, 210023, Jiangsu, China

^7^ School of Medicine and Holistic Integrative Medicine, Nanjing University of Chinese Medicine, Nanjing, 210023 Jiangsu, China

^8^ National Center of Colorectal Surgery, Nanjing Hospital of Chinese Medicine Affiliated to Nanjing University of Chinese Medicine, Nanjing, 210001, Jiangsu, China

^+^ Ya-ting Cao, Xin Huang, Cheng-li Yu and Jing Wang have contributed equally to this work.

^*^Corresponding author:

yal120_120@126.com (Ai-ling Yin) (ORCID iD: 0009-0005-7295-5011), zhangye_cpu@cpu.edu.cn (Ye Zhang) (ORCID iD: 0000-0001-5986-5971), fsyy00237@njucm.edu.cn (Kang Ding) (ORCID iD: 0000-0002-1524-6332).

**Table S1 Evaluation standard of colonic histological damage**

| **Histopathological index** |  | **Score 0** | **Score 1** | **Score 2** | **Score 3** | **Score 4** |
| --- | --- | --- | --- | --- | --- | --- |
| Tissue damage |  | Normal | Partial loss of goblet cells | Marked loss of goblet cells | Partial loss of crypts | Marked loss of crypts |
| Cell infiltration |  | Normal | Infiltration around crypt | Mucosal infiltration | Submucosal infiltration with mild edema | Muscularis layer with prominent edema |

**Table S2 Primer Sequences**

| Genes | Primer sequences（5’-3’） |
| --- | --- |
| *β-actin* | F:CTGTGCCCATCTACGAGGGCTAT |
|  | R:TTTGATGTCACGCACGATTTCC |
| *IL-6* | F:TAGTCCTTCCTACCCCAATTTCC |
|  | R:TTGGTCCTTAGCCACTCCTTC |
| *IL-1β* | F:GCAACTGTTCCTGAACTCAACT |
|  | R:ATCTTTTGGGGTCCGTCAACT |
| *Tnf-α* | F:CCCTCACACTCAGATCATCTTCT |
|  | R:GCTACGACGTGGGCTACAG |
| *IL-21* | F:GGACCCTTGTCTGTCTGGTAG |
|  | R:TGTGGAGCTGATAGAAGTTCAGG |
| *IL-22* | F:TGTGGAGCTGATAGAAGTTCAGG |
|  | R:GCTGGAAGTTGGACACCTCAA |
| *IL-17* | F:TCAGCGTGTCCAAACACTGAG |
|  | R:CGCCAAGGGAGTTAAAGACTT |
| *Dorea longicatena* | F:ATGACTGACGTTGAGGCTCG |
|  | R:GTTTACGGCGTGGACTACCA |
| *16s V3/V4* | F:CCTACGGGNGGCWGCAG |
|  | R:GACTACHVGGGTATCTAATCC |

**Table S3 Differential metabolites between the Control group and the DSS group**

| Metabolites | Rt/min | Mass charge ratio  (m/z) | VIP | *p* | FDR-*p* |
| --- | --- | --- | --- | --- | --- |
| Mannitol | 11.505 | 319.1817 | 1.67252 | 4.831E-08 | 7.633E-06 |
| 4-Oxoproline | 8.88 | 156.1012 | 1.656 | 3.425E-07 | 2.706E-05 |
| D-Tagatose | 11.107 | 307.1831 | 1.6415 | 1.017E-06 | 5.354E-05 |
| L-Tyrosine | 11.437 | 218.1036 | 1.62169 | 3.051E-06 | 1.205E-04 |
| Cholesterol | 18.149 | 129.011 | 1.61974 | 4.281E-06 | 1.353E-04 |
| Taurine | 9.882 | 326.1002 | 1.60242 | 8.081E-06 | 2.128E-04 |
| Glutamine | 10.449 | 156.1507 | 1.58407 | 2.017E-05 | 4.552E-04 |
| threo-hydroxyaspartic acid | 8.489 | 218.1239 | 1.58031 | 3.013E-05 | 5.952E-04 |
| allo-Inositol | 12 | 318.1939 | 1.53115 | 8.735E-05 | 1.533E-03 |
| L-Pipecolic acid | 7.747 | 156.1673 | 1.52637 | 1.060E-04 | 1.629E-03 |
| Palmitoleic acid | 11.815 | 311.2733 | 1.52 | 1.134E-04 | 1.629E-03 |
| N-methyl-L-glutamic Acid | 9.073 | 260.1803 | 1.49334 | 2.753E-04 | 3.346E-03 |
| Iminodiacetic acid | 8.521 | 232.1553 | 1.47699 | 4.121E-04 | 3.830E-03 |
| 2-Aminoadipic acid | 10.078 | 260.1437 | 1.46718 | 3.867E-04 | 3.826E-03 |
| D-Asparagine | 9.34 | 159.1461 | 1.45339 | 4.622E-04 | 4.057E-03 |
| 4-Hydroxynicotinamide | 4.964 | 152.0756 | 1.45192 | 5.361E-04 | 4.458E-03 |
| alpha-Tocopherol succinate | 17.794 | 237.1893 | 1.4457 | 5.875E-04 | 4.641E-03 |
| Cysteamine | 4.207 | 174.1418 | 1.44314 | 6.798E-04 | 5.115E-03 |
| Hydroxypyruvic acid | 4.919 | 147.076 | 1.43006 | 8.173E-04 | 5.870E-03 |
| cis-Caffeic acid | 8.529 | 219.1817 | 1.42471 | 8.810E-04 | 6.052E-03 |
| Indolelactic acid | 12.923 | 202.0816 | 1.38829 | 1.408E-03 | 9.216E-03 |
| Isopropyl-beta-D-thiogalactopyranoside | 11.626 | 361.1577 | 1.38728 | 1.458E-03 | 9.216E-03 |
| O-Phosphothreonine | 9.382 | 211.0331 | 1.36635 | 1.936E-03 | 1.177E-02 |
| Aspirin | 8.949 | 195.0825 | 1.365 | 2.307E-03 | 1.302E-02 |
| Pectin | 9.773 | 217.1613 | 1.35672 | 2.157E-03 | 1.262E-02 |
| 4-Hydroxystyrene | 6.916 | 192.1453 | 1.35182 | 2.595E-03 | 1.399E-02 |
| Sedoheptulose | 11.221 | 204.0534 | 1.3388 | 3.217E-03 | 1.588E-02 |
| Dehydroascorbic acid | 10.982 | 173.1308 | 1.3374 | 2.657E-03 | 1.399E-02 |
| Urea | 6.677 | 146.8743 | 1.32077 | 3.679E-03 | 1.661E-02 |
| Glutamic acid | 8.87 | 174.1366 | 1.32058 | 3.317E-03 | 1.588E-02 |
| Oxalacetic acid | 10.827 | 147.0789 | 1.31656 | 3.510E-03 | 1.631E-02 |
| Glucoheptonic acid | 11.496 | 333.1857 | 1.30941 | 4.481E-03 | 1.770E-02 |
| Gluconic acid | 11.723 | 333.1927 | 1.30498 | 3.800E-03 | 1.668E-02 |
| Glycolic acid | 5.287 | 147.0869 | 1.30322 | 3.986E-03 | 1.695E-02 |
| DL-Glutamate | 9.469 | 246.1503 | 1.30147 | 4.184E-03 | 1.695E-02 |
| Citric acid | 10.744 | 273.0937 | 1.29782 | 4.140E-03 | 1.695E-02 |
| 4-Methylumbelliferone | 4.754 | 248.1081 | 1.29329 | 4.805E-03 | 1.784E-02 |
| 2-Phenylacetamide | 4.05 | 88.08461 | 1.29034 | 4.600E-03 | 1.773E-02 |
| Tryptophyl-Tryptophan | 12.951 | 202.1168 | 1.28785 | 5.632E-03 | 1.894E-02 |
| D-Threitol | 8.748 | 217.1604 | 1.28724 | 4.874E-03 | 1.784E-02 |
| O-Phosphoethanolamine | 10.559 | 299.0995 | 1.28611 | 5.082E-03 | 1.784E-02 |
| Malic acid | 8.584 | 147.0901 | 1.28026 | 5.068E-03 | 1.784E-02 |
| Galactinol | 11.633 | 204.0339 | 1.2759 | 6.513E-03 | 2.100E-02 |
| myo-Inositol | 12.342 | 217.1132 | 1.27266 | 5.633E-03 | 1.894E-02 |
| Lysine | 11.324 | 174.1265 | 1.26 | 6.373E-03 | 2.098E-02 |
| diphosphate | 9.821 | 451.1116 | 1.23529 | 8.324E-03 | 2.579E-02 |
| 2-Hydroxybutanoic acid | 5.765 | 147.0955 | 1.22981 | 7.596E-03 | 2.401E-02 |
| Phthalic acid | 5.233 | 221.1266 | 1.22591 | 9.992E-03 | 2.871E-02 |
| Trans-3-hydroxy-L-proline | 10.459 | 274.1793 | 1.22169 | 9.965E-03 | 2.871E-02 |
| 2-Butyne-1,4-diol | 6.908 | 147.1005 | 1.22161 | 9.761E-03 | 2.871E-02 |
| Sulfuric acid 4-methoxyphenyl ester | 6.09 | 147.0438 | 1.214 | 9.957E-03 | 2.871E-02 |
| Lysylserine | 6.818 | 116.0962 | 1.16262 | 1.544E-02 | 4.356E-02 |
| Fumaric acid | 7.504 | 245.0983 | 1.14951 | 1.663E-02 | 4.608E-02 |

**Table S4 Differential metabolites between the DSS group and the QYD-HD group**

| Metabolites | Rt/min | Mass charge ratio  (m/z) | VIP | *p* | FDR-*p* |
| --- | --- | --- | --- | --- | --- |
| Taurine | 9.882 | 326.1002 | 1.39162 | 3.014E-05 | 4.762E-03 |
| Glutaminylglutamine | 10.449 | 156.1507 | 1.41167 | 7.319E-05 | 5.782E-03 |
| Sulfuric acid 4-methoxyphenyl ester | 6.09 | 147.0438 | 1.82176 | 2.134E-04 | 8.429E-03 |
| Dehydroascorbic acid | 10.982 | 173.1308 | 1.78248 | 2.404E-03 | 1.809E-02 |
| DOPA | 9.689 | 218.1155 | 1.75591 | 1.651E-03 | 1.739E-02 |
| Sedoheptulose | 11.221 | 204.0534 | 1.72916 | 2.021E-03 | 1.809E-02 |
| L-Tyrosine | 11.437 | 218.1036 | 1.68246 | 4.884E-04 | 1.212E-02 |
| allo-Inositol | 12 | 318.1939 | 1.68132 | 1.908E-03 | 1.809E-02 |
| 2-Phenylacetamide | 4.05 | 88.08461 | 1.66948 | 2.279E-03 | 1.809E-02 |
| Galactinol | 11.633 | 204.0339 | 1.64866 | 2.320E-03 | 1.809E-02 |
| Citric acid | 10.744 | 273.0937 | 1.62553 | 4.420E-03 | 2.817E-02 |
| Uracil | 7.511 | 241.1216 | 1.60814 | 1.462E-03 | 1.698E-02 |
| Melezitose | 15.465 | 361.1495 | 1.60192 | 6.134E-04 | 1.212E-02 |
| Indolelactic acid | 12.923 | 202.0816 | 1.59413 | 4.110E-03 | 2.817E-02 |
| 5-Aminopentanoic acid | 10.944 | 174.1488 | 1.58631 | 4.636E-03 | 2.817E-02 |
| L-Pipecolic acid | 7.747 | 156.1673 | 1.57384 | 5.633E-04 | 1.212E-02 |
| Methylglutamic acid | 9.073 | 260.1803 | 1.56369 | 1.505E-03 | 1.698E-02 |
| D-Alanyl-D-alanine | 9.427 | 188.1892 | 1.56048 | 1.053E-03 | 1.387E-02 |
| Conduritol epoxide | 4.133 | 147.0719 | 1.55729 | 5.681E-03 | 3.206E-02 |
| Iminodiacetic acid | 8.521 | 232.1553 | 1.51076 | 8.066E-03 | 4.353E-02 |
| beta-Alanine | 8.175 | 248.0861 | 1.4989 | 5.000E-04 | 1.212E-02 |
| 4-Hydroxynicotinamide | 4.964 | 152.0756 | 1.49712 | 4.875E-03 | 2.853E-02 |
| Cysteamine | 4.207 | 174.1418 | 1.49624 | 9.644E-03 | 4.915E-02 |
| [Hydroxypyruvic acid](https://hmdb.ca/metabolites/HMDB0001352) | 4.919 | 147.076 | 1.47984 | 2.308E-03 | 1.809E-02 |
| L-Valine | 6.529 | 144.1175 | 1.4256 | 8.265E-03 | 4.353E-02 |
| Dihydrouracil | 4.26 | 171.0288 | 1.40037 | 7.407E-04 | 1.233E-02 |

**Table S5 List of molecules (proteins) displayed in the heatmap**

| Uniprot accession number | Gene symbol | Protein name | Log2 fold change (DSS/Control) | Log2 fold change (Tyrosine/DSS) | Adjusted *p*-value (DSS/Control) | Adjusted *p*-value (Tyrosine/DSS) |
| --- | --- | --- | --- | --- | --- | --- |
| A0A075B5U0 | Ighv1-12 | Immunoglobulin heavy variable V1-12 | 2.0770761 | -1.7058366 | 0.00242088 | 0.039034481 |
| A0A075B684 | Ighv1-62-1 | Immunoglobulin heavy variable 1-62-1 | 3.3228632 | -2.2366452 | 0.001387896 | 0.033864179 |
| A0A0B4J1I8 | Igkv4-59 | Immunoglobulin kappa variable 4-59 (Fragment) | 2.4395504 | -2.1836279 | 0.014823291 | 0.046739057 |
| A0A0R4J0I1 | Serpina3k | Serine (or cysteine) peptidase inhibitor, clade A, member 3K | 3.2676653 | -2.7725601 | 0.008580077 | 0.047772852 |
| B1AR13 | Cisd3 | CDGSH iron-sulfur domain-containing protein 3, mitochondrial | -1.5562234 | 1.254767 | 0.001611091 | 0.032235705 |
| D3YWJ0 | Nuggc | Nuclear GTPase SLIP-GC | -0.9498183 | 0.8069723 | 0.007872156 | 0.041485709 |
| D3Z6Q9 | Bin2 | Bridging integrator 2 | 1.3741187 | -1.2361978 | 0.033673232 | 0.032235705 |
| G3UW81 | Cyp4f40 | Cytochrome P450, family 4, subfamily f, polypeptide 40 | -1.3748323 | 1.9255047 | 0.026207083 | 0.032235705 |
| G3X8T9 | Serpina3n | Serine (or cysteine) peptidase inhibitor, clade A, member 3N | 5.4362331 | -4.0026604 | 0.004577265 | 0.046739057 |
| G3X9Y6 | Akr1c19 | Aldo-keto reductase family 1, member C19 | -1.8975177 | 1.5596073 | 0.007753719 | 0.039034481 |
| O54890 | Itgb3 | Integrin beta-3 | 0.9067147 | -1.0976375 | 0.014201218 | 0.033941502 |
| O55100 | Syngr1 | Synaptogyrin-1 | 1.1437969 | -1.1049696 | 0.007948303 | 0.032235705 |
| O70475 | Ugdh | UDP-glucose 6-dehydrogenase | -1.6713696 | 1.3984224 | 0.023046106 | 0.047473204 |
| P00397 | Mtco1 | Cytochrome c oxidase subunit 1 | -1.9922201 | 1.8799099 | 0.011806638 | 0.039034481 |
| P04186 | Cfb | Complement factor B | 2.8412732 | -1.6001119 | 0.0006886 | 0.039765511 |
| P05555 | Itgam | Integrin alpha-M | 1.9116917 | -1.0994718 | 0.00169886 | 0.032235705 |
| P08226 | Apoe | Apolipoprotein E | 1.6081562 | -1.3476753 | 0.009291484 | 0.04793527 |
| P11835 | Itgb2 | Integrin beta-2 | 1.9135765 | -1.0475751 | 0.001965873 | 0.039034481 |
| P13597 | Icam1 | Intercellular adhesion molecule 1 | 1.9797438 | -1.6394665 | 0.001238563 | 0.026073003 |
| P14106 | C1qb | Complement C1q subcomponent subunit B | 2.6127127 | -1.1668157 | 0.00014899 | 0.040164542 |
| P19221 | F2 | Prothrombin | 1.5410964 | -0.8846479 | 0.018689202 | 0.039034481 |
| P21614 | Gc | Vitamin D-binding protein | 1.8510686 | -1.087427 | 0.003969993 | 0.039034481 |
| P1752 | Ap1g1 | AP-1 complex subunit gamma-1 | -0.4641476 | 0.3593761 | 0.034172884 | 0.047908779 |
| P26043 | Rdx | Radixin | 0.9627459 | -1.1897117 | 0.021377584 | 0.032235705 |
| P28667 | Marcksl1 | MARCKS-related protein | 2.8942183 | -2.5546576 | 0.007069364 | 0.03883683 |
| P29533 | Vcam1 | Vascular cell adhesion protein 1 | 2.2418674 | -0.9239922 | 0.000545766 | 0.032235705 |
| P30204 | Msr1 | Macrophage scavenger receptor types I and II | 1.5306091 | -0.8215966 | 0.001616589 | 0.039034481 |
| P30355 | Alox5ap | Arachidonate 5-lipoxygenase-activating protein | 2.7434414 | -1.5507782 | 0.000832269 | 0.041350117 |
| P30993 | C5ar1 | C5a anaphylatoxin chemotactic receptor 1 | 2.0813699 | -1.4339829 | 0.000785993 | 0.032235705 |
| P31725 | S100a9 | Protein S100-A9 | 6.9412934 | -2.1037322 | 7.11083E-05 | 0.039034481 |
| P35441 | Thbs1 | Thrombospondin-1 | 2.830927 | -2.2994612 | 0.004169078 | 0.033941502 |
| P48758 | Cbr1 | Carbonyl reductase [NADPH] 1 | -1.3614701 | 1.1493477 | 0.00169886 | 0.032235705 |
| P22892 | Sh3bp1 | SH3 domain-binding protein 1 | 1.3890832 | -0.7837876 | 0.001387896 | 0.041485709 |
| P58022 | Loxl2 | Lysyl oxidase homolog 2 | 1.5087452 | -1.253938 | 0.023895569 | 0.04793527 |
| P60202 | Plp1 | Myelin proteolipid protein | 1.1640839 | -1.1681231 | 0.01280691 | 0.039034481 |
| P61294 | Rab6b | Ras-related protein Rab-6B | 0.60158 | -0.6504382 | 0.0084238 | 0.026073003 |
| P70227 | Itpr3 | Inositol 1,4,5-trisphosphate-gated calcium channel ITPR3 | -0.6047856 | 0.5777443 | 0.028039368 | 0.026073003 |
| P97370 | Atp1b3 | Sodium/potassium-transporting ATPase subunit beta-3 | 0.7615319 | -0.7116183 | 0.035384933 | 0.039034481 |
| Q00519 | Xdh | Xanthine dehydrogenase/oxidase | 1.1497408 | -0.9355412 | 0.003778507 | 0.039034481 |
| Q03517 | Scg2 | Secretogranin-2 | 1.9779176 | -2.3235257 | 0.021855462 | 0.035994787 |
| Q03734 | Serpina3m | Serine protease inhibitor A3M | 4.6386006 | -3.6718064 | 0.002859195 | 0.039071605 |
| Q08481 | Pecam1 | Platelet endothelial cell adhesion molecule | 1.4081281 | -1.1884166 | 0.012132534 | 0.046739057 |
| Q09014 | Ncf1 | Neutrophil cytosol factor 1 | 2.0166242 | -1.3179978 | 0.03519322 | 0.039034481 |
| Q10470 | Mgat3 | Beta-1,4-mannosyl-glycoprotein 4-beta-N-acetylglucosaminyltransferase | -0.8255054 | 0.8183783 | 0.004447886 | 0.029090069 |
| Q3TBT3 | Sting1 | Stimulator of interferon genes protein | 1.5255945 | -0.9185422 | 0.005342293 | 0.041485709 |
| Q3TRM8 | Hk3 | Hexokinase-3 | 2.1731486 | -1.5732961 | 0.001925902 | 0.026073003 |
| Q3UIU2 | Ndufb6 | NADH dehydrogenase [ubiquinone] 1 beta subcomplex subunit 6 | -1.0756177 | 1.0139366 | 0.007110378 | 0.041485709 |
| Q3UMR5 | Mcu | Calcium uniporter protein, mitochondrial | -1.0355456 | 0.8274968 | 0.008453548 | 0.046739057 |
| Q3UZZ6 | Sult1d1 | Sulfotransferase 1 family member D1 | -3.0107747 | 1.4475873 | 0.003640356 | 0.048144209 |
| Q60590 | Orm1 | Alpha-1-acid glycoprotein 1 | 5.4454514 | -3.0124857 | 0.0006886 | 0.033410682 |
| Q60710 | Samhd1 | Deoxynucleoside triphosphate triphosphohydrolase SAMHD1 | 0.9663644 | -0.7668661 | 0.008453548 | 0.046739057 |
| Q60715 | P4ha1 | Prolyl 4-hydroxylase subunit alpha-1 | 1.4947291 | -1.0974676 | 0.003640356 | 0.04337479 |
| Q60963 | Pla2g7 | Platelet-activating factor acetylhydrolase | 2.2411423 | -2.3485123 | 0.013703762 | 0.046739057 |
| Q61147 | Cp | Ceruloplasmin | 3.7201299 | -1.9093979 | 0.000832269 | 0.039034481 |
| Q61646 | Hp | Haptoglobin | 6.7251909 | -2.2740999 | 0.003565653 | 0.039034481 |
| Q61703 | Itih2 | Inter-alpha-trypsin inhibitor heavy chain H2 | 2.5549979 | -1.2541621 | 0.000986708 | 0.04999541 |
| Q64435 | Ugt1a6 | UDP-glucuronosyltransferase 1-6 | -2.0416644 | 1.3617641 | 0.00691293 | 0.046739057 |
| Q64669 | Nqo1 | NAD(P)H dehydrogenase [quinone] 1 | -1.3577262 | 0.9044322 | 0.005806669 | 0.04999541 |
| Q68FL4 | Ahcyl2 | Putative adenosylhomocysteinase 3 | -1.1973063 | 0.9035834 | 0.011405889 | 0.039034481 |
| Q6ZQM8 | Ugt1a7 | UDP-glucuronosyltransferase 1A7 | -1.5976734 | 1.174744 | 0.005108817 | 0.030223736 |
| Q7TSJ2 | Map6 | Microtubule-associated protein 6 | 0.91555 | -1.5947305 | 0.047825426 | 0.032235705 |
| Q80XN0 | Bdh1 | D-beta-hydroxybutyrate dehydrogenase, mitochondrial | -1.7417623 | 1.3124556 | 0.00691293 | 0.041485709 |
| Q8BG07 | Pld4 | 5'-3' exonuclease PLD4 | 1.4751962 | -1.1904376 | 0.001387896 | 0.026073003 |
| Q8BH61 | F13a1 | Coagulation factor XIII A chain | 1.8197721 | -1.2754362 | 0.002317453 | 0.039765511 |
| Q8BKG3 | Ptk7 | Inactive tyrosine-protein kinase 7 | 1.2103121 | -0.9503791 | 0.008453548 | 0.046739057 |
| Q8BLX4 | Slc35c1 | GDP-fucose transporter 1 | -1.569913 | 1.6219153 | 0.018081194 | 0.046739057 |
| Q8BWM0 | Ptges2 | Prostaglandin E synthase 2 | -1.0897786 | 0.9512857 | 0.047270202 | 0.039034481 |
| Q8BX80 | Engase | Cytosolic endo-beta-N-acetylglucosaminidase | -1.1147333 | 1.0182285 | 0.005342293 | 0.032235705 |
| Q8BZS9 | Dhx32 | Putative pre-mRNA-splicing factor ATP-dependent RNA helicase DHX32 | -1.7043566 | 1.3227271 | 0.015918274 | 0.040164542 |
| Q8C0L0 | Tmx4 | Thioredoxin-related transmembrane protein 4 | 0.9800467 | -0.8256417 | 0.006902497 | 0.048241287 |
| Q8C0P5 | Coro2a | Coronin-2A | -0.7455517 | 0.7969023 | 0.025214056 | 0.046739057 |
| Q8CFB4 | Gbp5 | Guanylate-binding protein 5 | 2.0611486 | -1.4983339 | 0.000545766 | 0.04793527 |
| Q8CFX1 | H6pd | GDH/6PGL endoplasmic bifunctional protein | 1.449988 | -0.9842169 | 0.004675629 | 0.049685997 |
| Q8CG16 | C1ra | Complement C1r-A subcomponent | 1.8885537 | -1.2264414 | 0.01065465 | 0.035811075 |
| Q8K124 | Plekho2 | Pleckstrin homology domain-containing family O member 2 | 1.9407048 | -1.9766024 | 0.0018024 | 0.026073003 |
| Q8K3G9 | Appl2 | DCC-interacting protein 13-beta | -1.1975074 | 0.7568022 | 0.00311827 | 0.046739057 |
| Q8R138 | Tmem119 | Transmembrane protein 119 | 2.6146616 | -2.1916291 | 0.009939755 | 0.04793527 |
| Q8VCW8 | Acsf2 | Medium-chain acyl-CoA ligase ACSF2, mitochondrial | -1.9865664 | 1.3070371 | 0.005344622 | 0.041485709 |
| Q91VD9 | Ndufs1 | NADH-ubiquinone oxidoreductase 75 kDa subunit, mitochondrial | -1.0254754 | 0.5337288 | 0.000959036 | 0.046739057 |
| Q91WP6 | Serpina3n | Serine protease inhibitor A3N | 6.1135114 | -3.4204852 | 0.000785993 | 0.033410682 |
| Q91WV0 | Dr1 | Protein Dr1 | 1.5735525 | -1.6804338 | 0.02442402 | 0.032235705 |
| Q91X72 | Hpx | Hemopexin | 4.082766 | -2.3474436 | 0.001387896 | 0.039765511 |
| Q91XL1 | Lrg1 | Leucine-rich HEV glycoprotein | 4.6546712 | -2.4381631 | 0.001611091 | 0.046739057 |
| Q91Z40 | Gbp7 | Guanylate-binding protein 7 | 1.1160896 | -1.0715536 | 0.002254242 | 0.032235705 |
| Q923B6 | Steap4 | Metalloreductase STEAP4 | 3.7237704 | -1.5034406 | 0.000393036 | 0.040164542 |
| Q99K01 | Pdxdc1 | Pyridoxal-dependent decarboxylase domain-containing protein 1 | -1.9311913 | 1.3154139 | 0.004110841 | 0.046739057 |
| Q99KI0 | Aco2 | Aconitate hydratase, mitochondrial | -1.0362036 | 0.6183403 | 0.001904237 | 0.046739057 |
| Q99MN9 | Pccb | Propionyl-CoA carboxylase beta chain, mitochondrial | -1.0774471 | 0.5131128 | 0.004516562 | 0.035994787 |
| Q9CQW9 | Ifitm3 | Interferon-induced transmembrane protein 3 | 2.3845722 | -1.9644723 | 0.00169886 | 0.008082032 |
| Q9CRY7 | Gdpd1 | Lysophospholipase D GDPD1 | -1.548604 | 1.2755522 | 0.00169886 | 0.030223736 |
| Q9D1W4 | C030006K11Rik | RIKEN cDNA C030006K11 gene | 0.8805341 | -1.0832593 | 0.036460927 | 0.049424986 |
| Q9D289 | Trappc6b | Trafficking protein particle complex subunit 6B | 0.5244428 | -0.5766409 | 0.00691293 | 0.026073003 |
| Q9D2R0 | Aacs | Acetoacetyl-CoA synthetase | -1.1905724 | 0.9668311 | 0.007069364 | 0.033410682 |
| Q9D8P4 | Mrpl17 | Large ribosomal subunit protein bL17m | -0.8042483 | 0.6199847 | 0.004616255 | 0.040164542 |
| Q9DCC4 | Pycr3 | Pyrroline-5-carboxylate reductase 3 | -1.247507 | 0.9099927 | 0.004577265 | 0.032235705 |
| Q9DCE9 | Igtp | Immunity-related GTPase family M protein 3 | 1.5783341 | -1.8102582 | 0.009191912 | 0.032235705 |
| Q9DCM2 | Gstk1 | Glutathione S-transferase kappa 1 | -1.1597842 | 1.1048701 | 0.017070894 | 0.047596073 |
| Q9DCS1 | Tmem176a | Transmembrane protein 176A | 2.6851416 | -1.7176361 | 0.011405889 | 0.046739057 |
| Q9ES64 | Ush1c | Harmonin | -0.8565311 | 0.8999076 | 0.034226974 | 0.032235705 |
| Q9JHF5 | Tcirg1 | V-type proton ATPase subunit a | 1.3584212 | -0.8825521 | 0.000832269 | 0.032235705 |
| Q9JIK9 | Mrps34 | Small ribosomal subunit protein mS34 | -1.0388772 | 1.0567108 | 0.004353869 | 0.026073003 |
| Q9JK92 | Hspb8 | Heat shock protein beta-8 | 1.5231278 | -1.6026341 | 0.022225682 | 0.032235705 |
| Q9JMG2 | C1galt1c1 | C1GALT1-specific chaperone 1 | -1.9984117 | 1.3667611 | 0.004169078 | 0.025985599 |
| Q9QXC1 | Fetub | Fetuin-B | 2.884648 | -1.7999561 | 0.00169886 | 0.032235705 |
| Q9QXG4 | Acss2 | Acetyl-coenzyme A synthetase, cytoplasmic | -1.3412749 | 0.9902283 | 0.005180472 | 0.045413693 |
| Q9QXS6 | Dbn1 | Drebrin | 1.3275793 | -1.2370974 | 0.046284173 | 0.04337479 |
| Q9QZS5 | Sgk2 | Serine/threonine-protein kinase Sgk2 | 1.0004146 | -0.9095541 | 0.021931422 | 0.041485709 |
| Q9R0B9 | Plod2 | Procollagen-lysine,2-oxoglutarate 5-dioxygenase 2 | 2.3374836 | -2.0221666 | 0.041051713 | 0.041485709 |
| Q9R233 | Tapbp | Tapasin | 0.7915014 | -1.075774 | 0.022158384 | 0.047772852 |
| Q9WTS2 | Fut8 | Alpha-(1,6)-fucosyltransferase | -0.8778518 | 0.9502637 | 0.043447828 | 0.04793527 |
| Q9Z0E6 | Gbp2 | Guanylate-binding protein 2 | 1.7175343 | -1.7175343 | 0.000239531 | 0.008082032 |


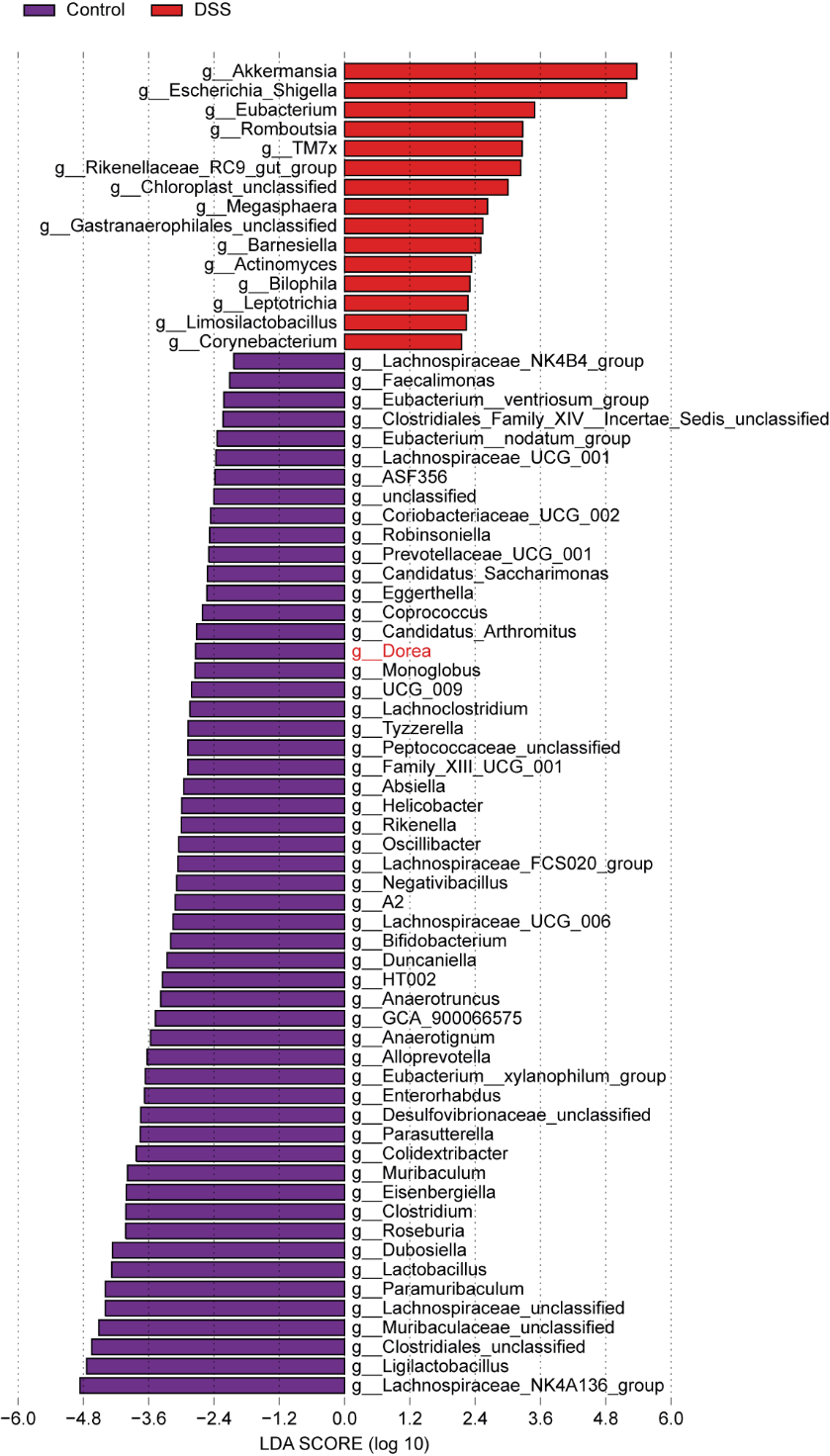


**Figure S1**: LEfSe analysis revealing significantly different bacterial communities between Control and DSS groups (LDA > 2).


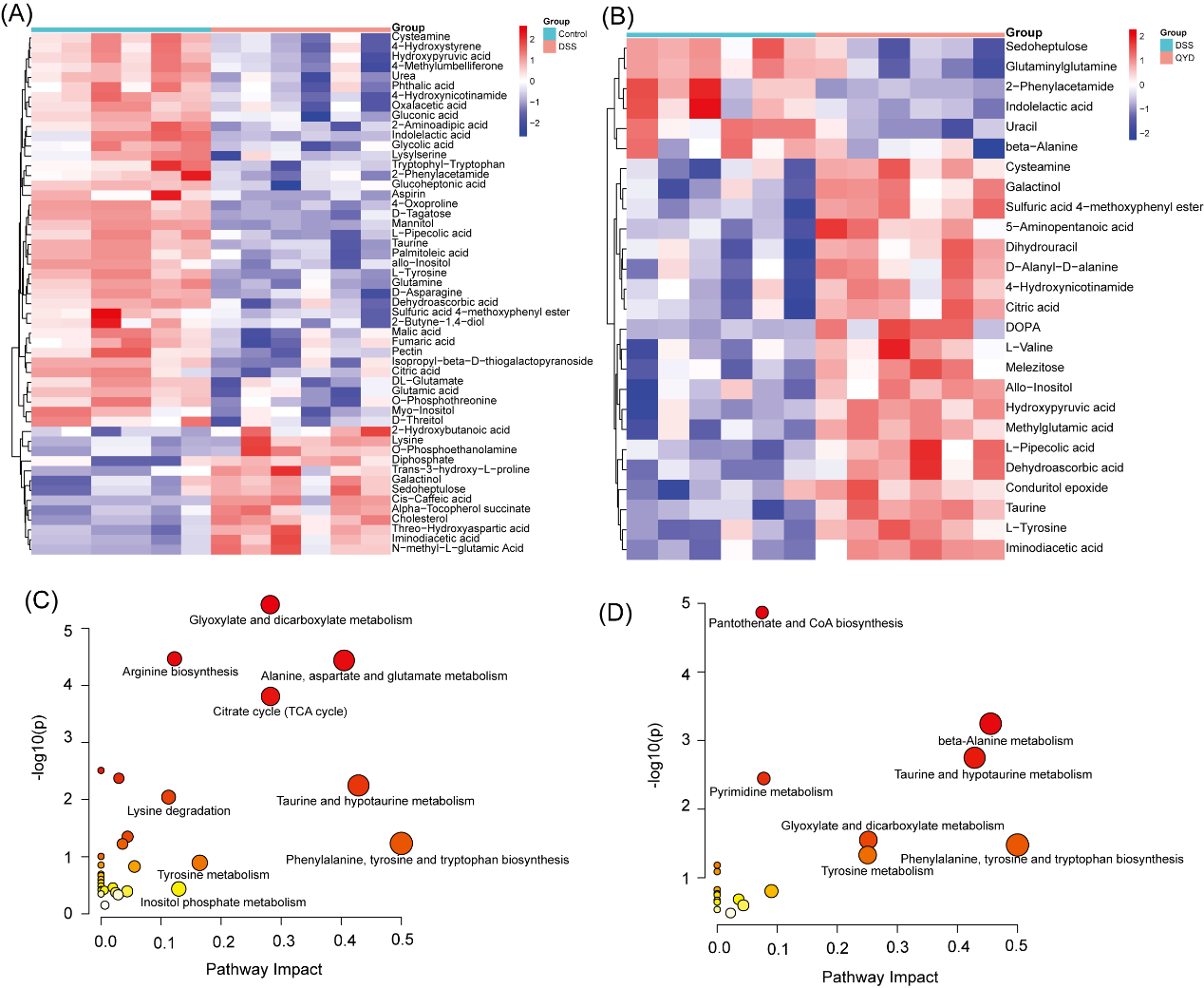


**Figure S2**: (A) Differential Metabolite Heatmap Between Control and DSS Groups. (B) Differential Metabolite Heatmap Between DSS and QYD-HD Groups. (C) Differential Metabolites Between Control and DSS KEGG Pathway Enrichment Plot. (D) Differential Metabolites Between DSS and QYD-HD KEGG Pathway Enrichment Plot.
